# Macrophage migration inhibitory factor (MIF) and the tumor ecosystem: a tale of inflammation, immune escape, and tumor growth

**DOI:** 10.3389/fimmu.2025.1636839

**Published:** 2025-10-13

**Authors:** Rana A. Youness, Noha M. Elemam, Abdelhamid M. Abdelhamid, Adham H. Mohamed, Lolowa M. Elsherbiny, Asmaa Ramzy, Reem A. Assal

**Affiliations:** ^1^ Molecular Biology and Biochemistry Department, Molecular Genetics Research Team (MGRT), Faculty of Biotechnology, German International University (GIU), Cairo, Egypt; ^2^ Clinical Sciences Department, College of Medicine, University of Sharjah, Sharjah, United Arab Emirates; ^3^ Research Institute for Medical and Health Sciences, University of Sharjah, Sharjah, United Arab Emirates; ^4^ Biotechnology School, Nile University, Giza, Egypt; ^5^ Department of Chemistry (Biochemistry Division), Faculty of Science, Cairo University, Giza, Egypt; ^6^ Proteomics and Metabolomics Research Program, Basic Research Unit, Research Department, Children’s Cancer Hospital Egypt, Cairo, Egypt; ^7^ Department of Pharmacology and Toxicology, Heliopolis University for Sustainable Development (HU), Cairo, Egypt

**Keywords:** macrophage migration inhibitory factor (MIF), immune regulation, solid tumors, inflammatory mediator, tumor microenvironment

## Abstract

Macrophage migration inhibitory factor (MIF) is a pleiotropic cytokine with a pivotal role in immune regulation, inflammation, and tumorigenesis. Originally identified as a T cell-derived factor inhibiting macrophage migration, MIF has since been recognized as a key player in the progression of a wide range of solid tumors. This comprehensive review traces the historical discovery and evolving understanding of MIF, highlighting its structural features, receptor interactions, and intracellular signaling mechanisms. The review also explores the molecular mechanisms of MIF involvement in tumor pathogenesis through promoting proliferation, angiogenesis, immune evasion, and metastasis. Special focus is given to MIF interplay with several oncogenic pathways, modulation of the tumor microenvironment, and its dual role in both autocrine and paracrine signaling within tumors. The review also discusses emerging insights into MIF’s involvement in therapeutic resistance and its potential as a diagnostic biomarker and therapeutic target. By consolidating current knowledge, the authors aim to provide a detailed perspective on MIF’s multifaceted role in solid tumors and to outline future directions for research and clinical intervention.

## Graphical Abstract

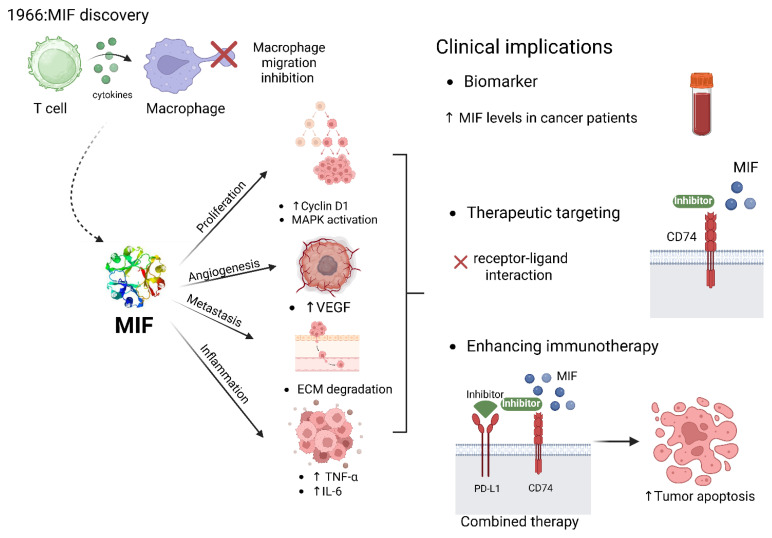

## 1 Introduction

**Figure 1:**
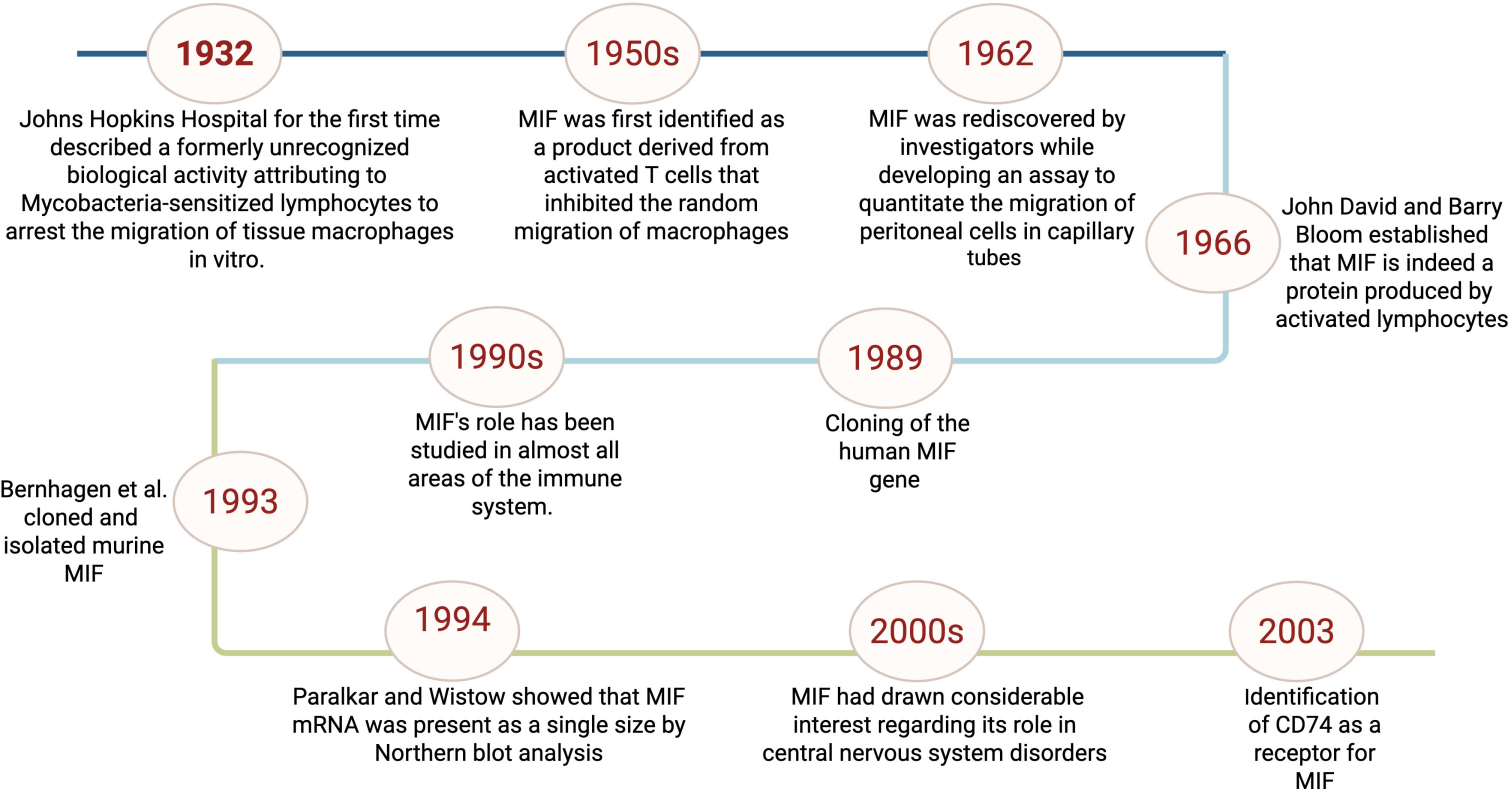
Timeline of key discoveries related to macrophage migration inhibitory factor (MIF) This figure represents a schematic representation for the timeline of MIF initial identification to the discovery of its receptor and roles in immunity.

Macrophage migration inhibitory factor (MIF) is a multi-functional cytokine that exerts a crucial role in immune and inflammatory regulation (1). It is recognized as a pro-inflammatory cytokine and hormone that performs vital roles in stress responses. MIF is primarily produced in the anterior pituitary gland; its secretion is induced by corticotrophin-releasing hormone in response to stress (2). MIF is secreted by various cells including monocytes, macrophages, B cells, T cells, in addition to endocrine, endothelial, and epithelial cells (3) and is released by binding of toll-like receptors or pattern recognition receptors to small molecular motifs such as pathogen-associated molecular patterns (4). On the functional level, MIF is involved in a variety of biological functions including the production of inflammatory cytokines, such as tumor necrosis factor, interleukin-6, interferon-γ, and interleukin-1β; hormone immunomodulation; muscle glucose catabolism regulation; tumor growth promotion; and pathology of diseases such as rheumatoid arthritis, asthma, lupus, and atherosclerosis (5). Moreover, MIF exhibits chemokine-like activity besides stimulating target cell migration and recruiting leukocytes to infectious and inflammatory areas (6). The MIF journey, starting from its discovery to its current role as one of the pivotal cytokines in immunology, represents a remarkable odyssey that traces the evolution of concepts on immune regulation as illustrated in **Figure 1**. In 1932, the Bulletin of the Johns Hopkins Hospital described a formerly unrecognized biological activity attributing to Mycobacteria-sensitized lymphocytes to arrest the migration of tissue macrophages *in vitro* (7). In the late 1950s, MIF was first identified as a product derived from activated T cells that inhibited the random migration of macrophages which was related to delayed-type hypersensitivity reactions (8). In 1962, MIF was identified to quantitate the migration of peritoneal cells in capillary tubes (9). Independently, in 1966, John David and Barry Bloom reported that MIF is indeed a protein produced by activated lymphocytes further establishing MIF as a unique cytokine (10). Cloning of the human MIF gene in 1989 provided information about its structure, biological roles, and functional features (11).

In 1996, the three-dimensional crystal structure of MIF was identified unraveling a novel protein fold and nominating MIF as a new superfamily. This structural insight suggested that the natural form of MIF is a homotrimer with a molecular weight of about 37.5 kDa, thus providing a structural basis for understanding its functional properties (12).

**Figure 2:**
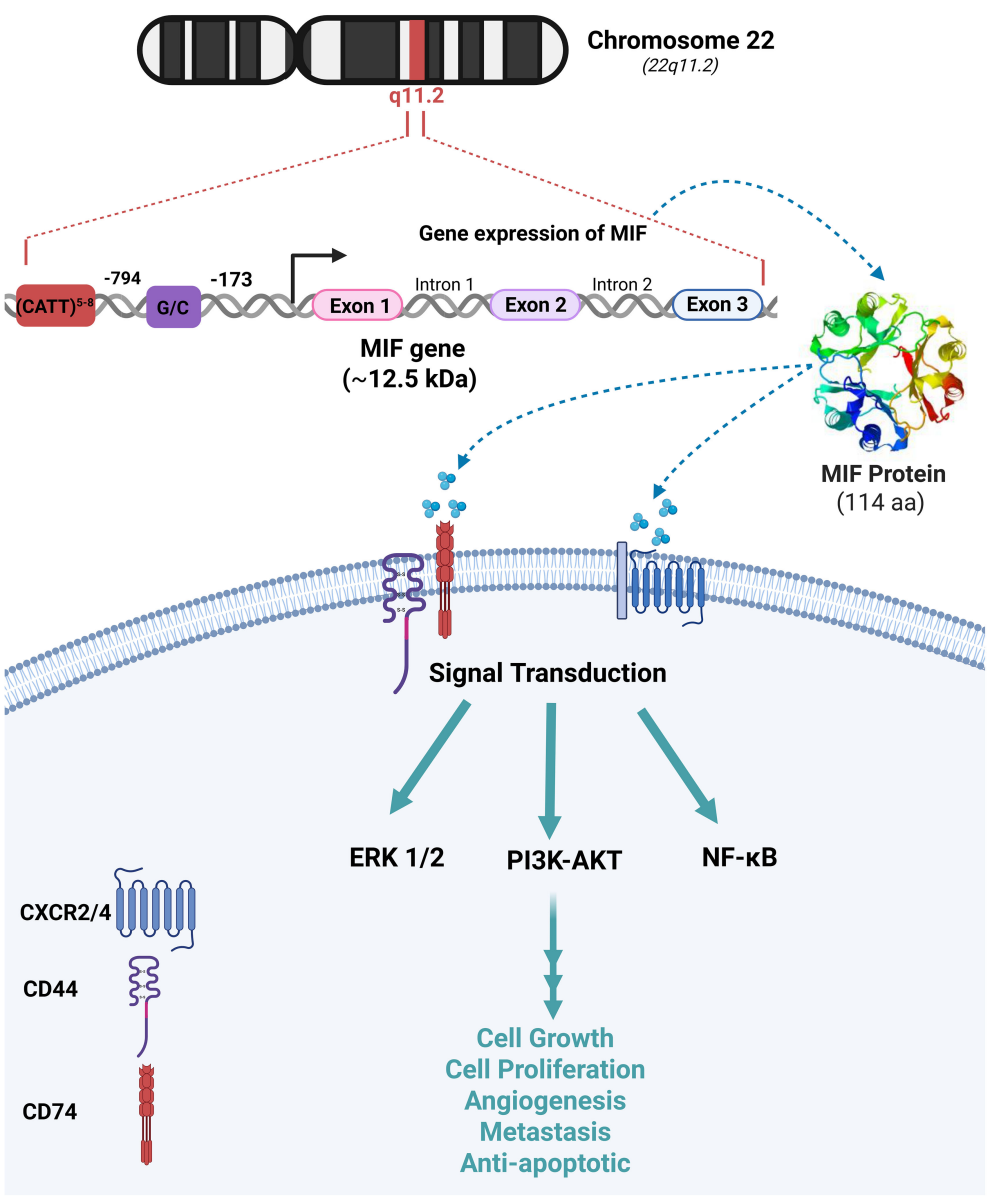
MIF Chromosomal location, genetic dissection and biological functions The migration inhibitory factor (MIF) gene is located on chromosome 22q11.2 and consists of three exons separated by two introns. Its transcription is regulated by two distinct promoter elements, CATT5–8 and G/C, positioned at nucleotides 794 and 173, respectively. MIF exerts its biological functions through interactions with CD74, CD44, and chemokine receptors CXCR2/4 on the cell surface. These interactions activate downstream signaling cascades, including the extracellular signal-regulated kinase 1/2 (ERK1/2), phosphatidylinositol 3-kinase/protein kinase B (PI3K/AKT), and nuclear factor kappa B (NF-κB) pathways. These pathways regulate key cellular processes such as proliferation, growth, angiogenesis, metastasis, and anti-apoptosis.

Human MIF depicts 90% sequence similarity with mouse MIF. MIF gene is located on chromosome 22 (22q11.2) and consists of three short exons and two introns. Besides, two promoter polymorphisms -CATT5–8 and G/C- are located at positions –794 and –173, respectively (**Figure 2**) (13). MIF encodes an evolutionarily conserved protein with a relative molecular mass of ~12.5 kDa, composed of 114–115 amino acids folded into an enzymatically active homotrimer with four β-strands and two α-helices (14). The catalytic function of MIF is attributed to a distinctive N-terminal proline that enables the keto-enol tautomerization of substrates such as D-dopachrome and L-dopachrome methyl ester into their corresponding indole derivatives (15). CD74 is a type II transmembrane protein with an apparent molecular weight of 31–41 kDa (16). MIF interacts with the extracellular domain of CD74 transmembrane receptor, resulting in the activation of various signaling pathways such as Extracellular Signal-Regulated Kinase (ERK), Phosphatidylinositol 3-Kinase (PI3K)-Akt, and Nuclear Factor-kappa B (NF-κB) (16). The MIF-induced cellular functions are mostly mediated by mitogen-activated protein kinases (MAPKs) (16). MIF-dependent phosphorylation of extracellular signal-regulated kinases (ERK-1/2), synthesis of prostaglandin E2, and cell proliferation rely on the cell surface expression of CD74 (6). The cytoplasmic tail of CD74 lacks a signal transduction domain; however, CD44 constitutes an essential part of the CD74 receptor complex through which MIF signal transduction occurs. The interaction of MIF with this receptor complex phosphorylates CD74 and CD44 in the intracytoplasmic domain (17). CD74 is essential for MIF-mediated protection against apoptosis, which requires the presence of its receptor counterpart, CD44. The polymorphic transmembrane protein CD44 has demonstrated tyrosine kinase activation characteristics and activates Src-family tyrosine kinases which in turn phosphorylate ERK (**Figure 2**) (17). In addition, MIF directly interacts with the chemokine receptors CXCR2 and CXCR4, modulating cell migration and inflammation by activating the ERK1/2 and PI3K/Akt signaling pathways. MIF rapidly associates with CXCR2 and competes with its ligands (18), which suggests that MIF signaling pathway is mediated by the complex CD74/CXCR2 receptor (**Figure 2**) (19).

## 2 Intracellular MIF mechanisms in innate immunity, inflammation, and autoimmunity

MIF interacts with intracellular proteins such as c-Jun activation domain-binding protein 1 (JAB1) which is a coactivator of the Activator Protein 1 (AP-1) transcription factor (20). The MIF–JAB1 interaction inhibits the degradation of cyclin-dependent kinase inhibitor p27Kip1 (21), which in turn results in cell cycle arrest (22). MIF exerts control over its functions through catalytic activities (23), where it exhibits both enzymatic thiol-protein oxidoreductase and tautomerase/isomerase activities (24), which are implicated in the catecholamine-converting activity of MIF (25). We will further discuss the critical roles of MIF in modulating the immune system.

**Figure 3:**
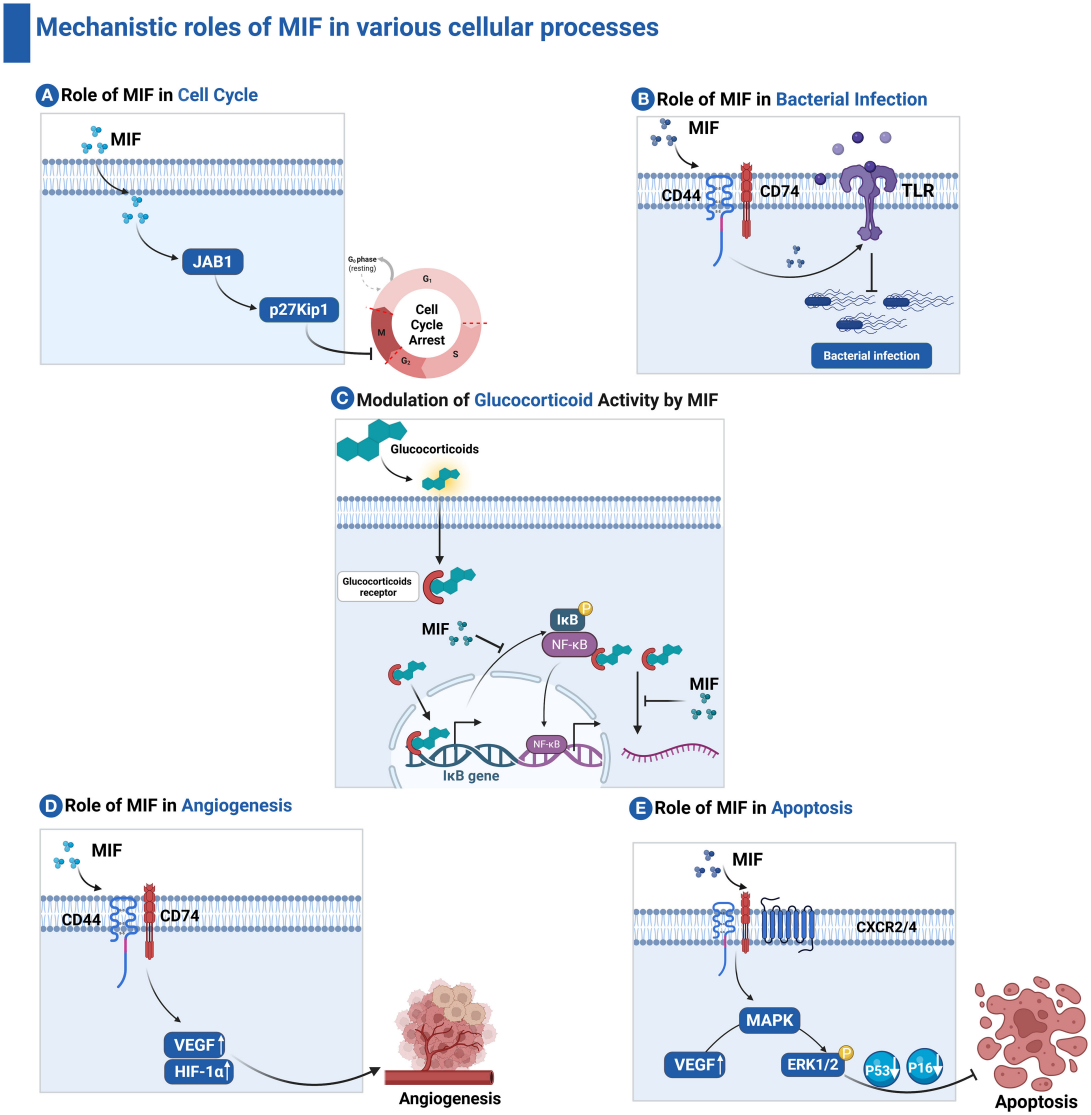
Mechanistic roles of MIF in various cellular processes. **(A)** Role of MIF in Cell Cycle: MIF interacts with JAB1, preventing the degradation of the cyclin-dependent kinase inhibitor p27Kip1, thereby inducing cell cycle arrest. **(B)** Role of MIF in bacterial infection: MIF promotes the expression of pro-inflammatory cytokines and upregulates TLR4, enhancing bacterial recognition. **(C)** Modulation of Glucocorticoid Activity by MIF: MIF antagonizes the immunosuppressive effects of glucocorticoids by inhibiting glucocorticoid-induced IκB synthesis and mRNA destabilization. **(D)** Role of MIF in Angiogenesis: MIF engages CD74 and CD44 receptors to promote the release of vascular endothelial growth factor (VEGF), thereby facilitating angiogenesis. **(E)** Role of MIF in apoptosis: MIF activates ERK1/2 signaling, leading to suppression of pro-apoptotic proteins such as p53 and p16, ultimately inhibiting apoptosis.

MIF has a crucial role in the regulation of innate immunity (26, 27). The cytokine is constitutively expressed by most immune cells, including macrophages, and is rapidly released in response to stress and microbial stimulation (28). MIF induces the expression of pro-inflammatory cytokines and up-regulates TLR4, thus facilitating the recognition of bacterial infections such as *Salmonella typhimurium* (29). MIF triggers several signal pathways, including the activation of the mitogen-activated protein kinase-analyzed ERK1/ERK2 pathway, necessary for macrophage activation and function (17). It further suppresses p53-dependent apoptosis in macrophages to allow continuous execution of pro-inflammatory functions as shown in **Figure 3** (30). MIF is implicated in numerous inflammatory and autoimmune conditions, such as rheumatoid arthritis, septic shock, and inflammatory bowel disease. Its dysregulation can lead to exacerbation of the inflammatory response and perpetuation of diseases (31). Furthermore, it plays a pivotal role in the modulation of inflammatory response. By activating immune cells through its receptor CD74, MIF modulates the secretion of pro-inflammatory cytokines, such as TNF-α, IL-1β, and IL-6 (32). This pro-inflammatory activity is crucial in response to infection and injury, helping to coordinate defense mechanisms (33). However, in chronic inflammatory conditions, sustained MIF expression can contribute to pathological inflammation, leading to tissue damage and autoimmune diseases.

One of the hallmarks and unique characteristics of MIF is its ability to counteract the immunosuppressive action of glucocorticoids, thus preserving the immunological responses during conditions of inflammation and stress and allowing a much more effective immune response against pathogens (34). Furthermore, MIF promotes the migration and recruitment of immune cells by inducing the expression of chemokines and adhesion molecules as shown in **Figure 3**. These functions are crucial for appropriate immune surveillance and infection responses (35). Recently, MIF has been implicated in tumor immunity; it can establish both pro-inflammatory and immunosuppressive environments depending on the context. MIF has a dual role in tumor development through modulating tumor growth and angiogenesis (36). It promotes angiogenesis, which is critical for tumor growth and metastasis, through enhancing the release of vascular endothelial growth factor, a key mediator of angiogenesis as shown in **Figure 3**. This role is essential not only in the context of wound healing and tissue regeneration but also in enabling tumors to establish their blood supply, facilitating their growth and metastasis (37–39).

## 3 Interactions of MIF with immune cells in the tumor microenvironment

MIF acts on a wide array of immune cells, including macrophages, T cells, dendritic cells, Tumor-Associated Neutrophils (TANs), Myeloid-Derived Suppressor Cells (MDSCs), and natural killers’ cells (40). The interaction with all these elements has a wide-ranging effect on tumor development, and immune evasion.

### 3.1 Macrophages

**Figure 4:**
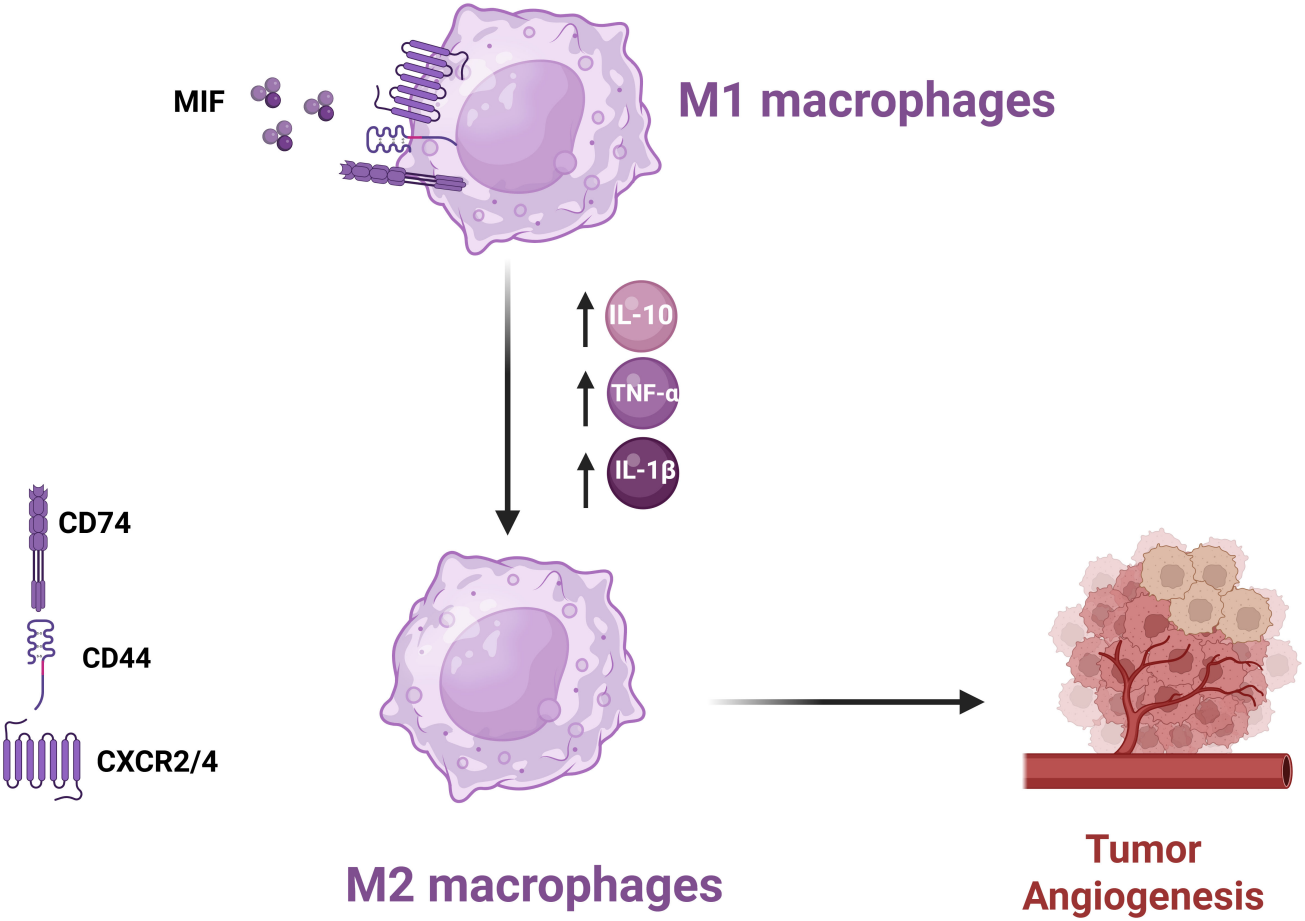
*Mechanism of MIF-Induced Polarization of M1 Macrophages to M2 Phenotype* MIF promotes M2 macrophage polarization in the TME, enhancing angiogenesis and suppressing anti-tumor immune responses, thereby facilitating tumor progression.

In the context of TME, MIF plays a very critical role in polarizing macrophages toward an M2 immunosuppressive and tissue repair phenotype (27, 41). Polarization mediates tumor growth due to enhanced tumor angiogenesis and suppression of anti-tumor immunity (42). MIF acts mainly through its receptor CD74 together with co-receptors, including CD44 and CXCR4. These complexes, upon binding with MIF, trigger the signaling pathways that culminate in the release of various cytokines and chemokines to promote an immunosuppressive milieu (43). The activation of NF-κB mediates transcription of various pro-inflammatory cytokines, including IL-10. MIF is able to increase the secretion of IL-10 from macrophages and offers a more permissive immunological milieu. Besides IL-10, MIF can induce the release of other pro-inflammatory cytokines like TNF-α and IL-1β. This dual role of stimulating pro-inflammatory cytokines while enhancing anti-inflammatory signals underlines the very complex nature of MIF influence on macrophage function within the TME (**Figure 4**) (43).

### 3.2 T cells

**Figure 5:**
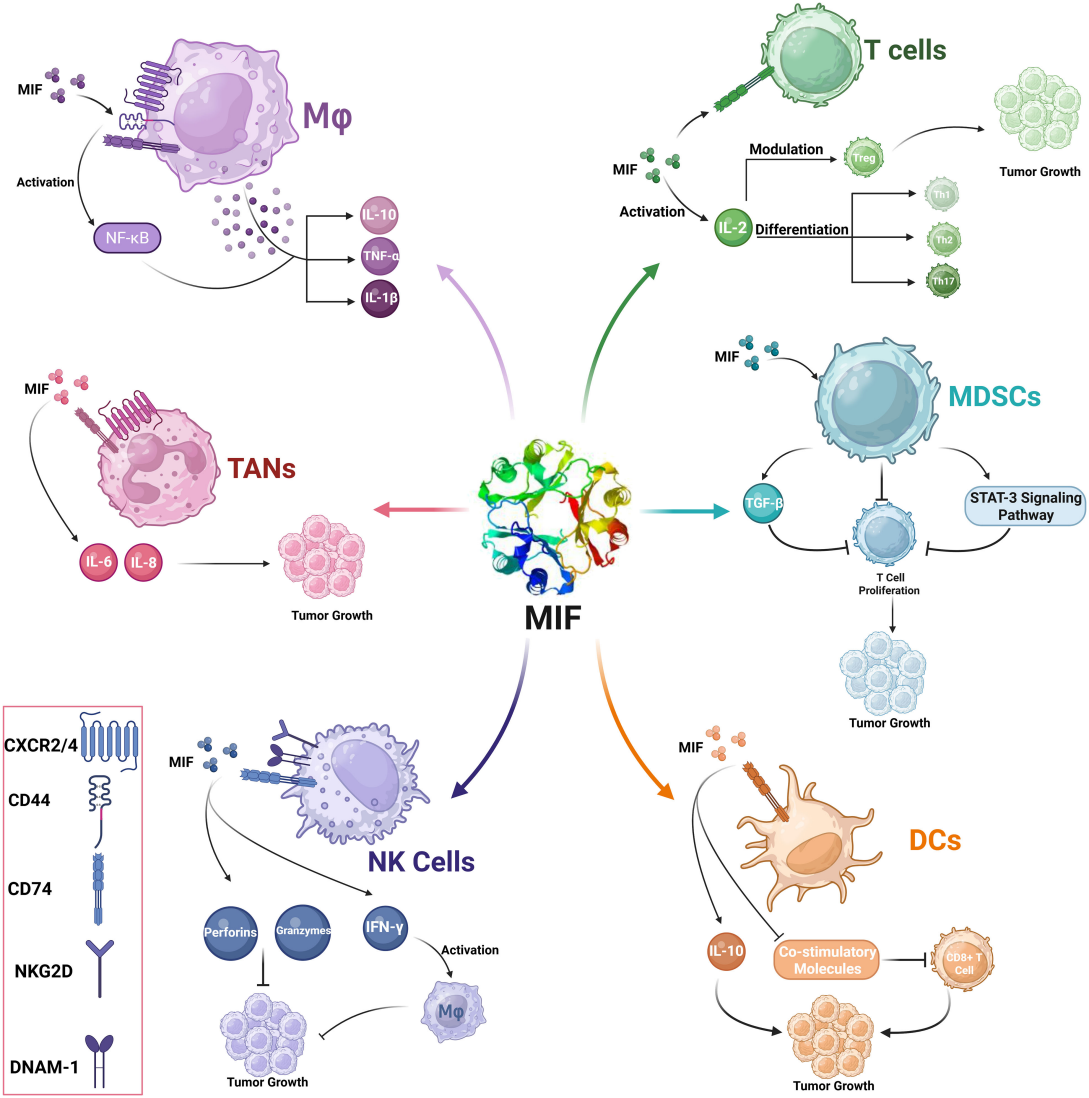
MIF-mediated modulation of immune cells in the tumor microenvironment (TME). This schematic illustration represents the role of MIF in modulating several immune cells at the TME.

MIF has a substantial impact on T cell functionality by influencing the activation and differentiation of numerous T cell subsets, including Th1, Th2, and Tregs (41, 44). High levels of MIF can cause immunosuppressive phenotypes, which prevent efficient anti-tumor responses. Previous studies have demonstrated that MIF stimulates the development of Tregs by modulating IL-2 production, hence increasing tumor growth by generating an environment permissive to immunological tolerance (45). CD74 is expressed on the surface of activated T lymphocytes, and its binding to MIF causes important intracellular signaling events. This involves the stimulation of pathways involved in T cell migration 3and proliferation. MIF’s interaction with CD74 increases Th17 differentiation, supporting a more aggressive immune response in some circumstances and contributing to T cell exhaustion in others. Of note, MIF increases Treg activities; thus, MIF increases the formation of tumor-associated Tregs that are crucial in maintaining immunological tolerance in the TME. Notably, MIF knockout models demonstrated a high reduction in Treg levels and increased anti-tumor immunity (**Figure 5**) (19, 45).

### 3.3 Dendritic cells

MIF controls the function and maturation of DCs, which represent one of the most powerful antigen-presenting cells of the immune system. Through controlling the interaction of DC with T cells, MIF alters the adaptive immune response toward tumors. The major receptor through which MIF acts on DCs is the CD74 receptor, which represents the invariant chain of MHC class II molecules (46). The interaction plays an important role in triggering downstream signaling pathways that control DC activity. MIF binding to CD74 suppresses the expression of key co-stimulatory molecules on DCs. This downregulation inhibits T-cell activation and proliferation. MIF acts through manipulating the cytokine profile that is produced by DCs. Instead of secreting pro-inflammatory cytokines such as IL-12, which induce Th1 responses, MIF-exposed DCs may secrete immunosuppressive cytokines such as IL-10. This further contributes to the tolerogenic environment that allows tumor development (**Figure 3**) (47).

MIF interaction with CD74 triggers a number of intracellular signaling pathways, including the PI3K/Akt pathway facilitates cell survival, but it may also have immunosuppressive implications when triggered in DCs. Activated ERK1/2 can lead to long-term inflammatory reactions, but upon being influenced by MIF, it also expresses the capability to induce tolerance. Binding of MIF to CD74 downregulates key co-stimulatory molecule expression in DCs. This results in impaired efficient T cell activation and proliferation (**Figure 5**) (48).

The elevated levels of MIF may result in poor maturation and functioning of DCs, thereby affecting their capability for activation of CD8+ cytotoxic T lymphocytes (49). Mature DCs generally deliver antigens in an efficient manner and provide the co-stimulatory cues necessary for the activation of CTLs. However, where maturation of DCs is impeded by MIF, owing to the inability to effectively present tumor antigens, the activation of CTL cells is lowered, reducing anti-tumor immunity. Poor effector function combined with increased expression of inhibitory receptors, such as PD-1, is characteristic of T cell exhaustion driven by chronic exposure to an immunosuppressive environment. If T cell responses can be downregulated and blocked, then tumors may escape immune surveillance eventually leading to tumor progression and spread. In conclusion, MIF regulates the production of immune suppressive factors by DCs and other immune cells that promote tumor growth (48, 49).

### 3.4 Natural killer cells

MIF modulates the complex activities of NK cells, one of the primary elements of the innate immune response that directly kill tumor cells (50, 51). Most of MIF functions are mediated with CD74, which is expressed on NK cells (52). The interaction induces downstream signaling that includes PI3K/Akt and MAPK/ERK cascades, which are required for activation and proliferation of NK cells (51, 53, 54). These pathways result in the increased synthesis of cytotoxic chemicals such as perforins and granzymes, which enhance the lysing capability of NK cells against tumor cells (55). MIF can induce NK cells to produce pro-inflammatory cytokines, including IFN-γ. IFN-γ, in turn, activates macrophages and enhances Th1 responses, further augmenting the antitumor immune response. MIF interacting with its receptors on NK cells upregulates the cytotoxic activity of NK cells. This includes the overexpression of activating receptors such as natural killer group 2D (NKG2D) and DNAX accessory molecule-1 (DNAM-1), which is needed for recognition and killing of tumor cells (**Figure 5**). The high levels of MIF in TME may evoke an immunosuppressive form leading to lower NK cell cytotoxicity, as high levels of MIF inhibit the NK cell-activating receptors, thus limiting its tumor cell recognition and killing capability (40). Furthermore, high levels of MIF have been associated with transcriptional down-regulation of NKG2D on the NK cells, hence impeding its cytotoxic activity against the tumor cells (56).

### 3.5 Myeloid-derived suppressor cells

Myeloid-Derived Suppressor Cells (MDSCs) are generally divided into two subsets, namely monocytic (M-MDSCs) and granulocytic or polymorphonuclear (PMN-MDSCs). They are characterized by their capability of inhibiting T cell proliferation and promoting Tregs expansion, thus contributing to an immune suppressive environment that favors tumor growth (57). MIF significantly influences the recruitment and expansion of MDSCs in the TME. Clinical evidence has shown that high levels of MIF are accompanied by higher levels and activity of MDSCs, an integral aspect of tumor immune survival. MIF promotes the expansion of myeloid cells into MDSCs (58). Several reports have identified that tumor-derived MIF can drive the expansion of both monocytic and granulocytic populations, thus enhancing the overall immunosuppressive capability of the tumor. MIF also enhances the production of key immunosuppressive molecules by MDSCs including Arginase-1 which lowers the levels of L-arginine in the microenvironment, thus directly inhibiting T cell activation (**Figure 5**) (59).

MIF triggers the STAT3 signaling pathway, which is involved in MDSC differentiation and function. Activation of STAT3 upregulates the production of various immunosuppressive molecules and promotes the capability to impede T cell responses from MDSCs. This mechanism also maintains the immunosuppressive phenotype in these cells, which enables their survival within the TME (60). MIF also acts by triggering the production of TGF-β by MDSCs. TGF-β is an immunosuppressive cytokine. It suppresses the function of effector T cells while promoting the development and proliferation of Tregs in parallel. TGF-β is present in the TME, maintaining or enhancing the immunosuppressive environment that enables tumors to escape immune recognition. The interaction between MIF and its receptor CXCR2 on MDSCs induces the migration of MDSCs toward the tumor sites. This causes a chemotactic effect that can increase the concentration of suppressor cells within the TME (**Figure 3**) (61).

### 3.6 Tumor-associated neutrophils

Upon infiltrating the TME, Tumor-Associated Neutrophils (TANs) represent one subpopulation of neutrophils and express either pro- or anti-tumor activities. The interaction between TANs and MIF influences TAN activities and tumor progression, structuring the immune compartment of TME (62). MIF promotes neutrophil infiltration into tumors as a chemotactic factor. This is further facilitated by the interaction with receptors like CXCR2 on TANs, thereby favoring recruitment of the latter within the TME (18).

MIF and TANs communicate through several signaling pathways, which comodulate their functions. MIF binds to CD74 on neutrophils, thereby activating downstream signaling cascades that promote cell survival, proliferation, and activation. The activation of pathways such as ERK1/2 and AKT due to this interaction leads to an increased expression of pro-tumoral genes. MIF may change the cytokine secretion profile of TAN. It could induce the release of IL-6 and IL-8, which would attract more immune cells to the TME and accelerate inflammatory responses that support tumor growth (**Figure 5**) (46, 63).

## 4 Role of MIF in immune cells and inflammation

MIF plays a role in various biological functions, such as leukocyte recruitment, inflammation, immune responses, cell proliferation, tumor development, and the counter-regulation of glucocorticoids in both physiological and pathological processes. Previous studies reported MIF’s role in inflammatory and immune mediated diseases such as rheumatoid arthritis, cancer, multiple sclerosis, systemic lupus erythematosus (SLE), and psoriasis (64–68). A possible pathogenic mechanism is through the enhanced production of inflammatory molecules such as TNF-α, nitric oxide, IL-1, IL-6, IL-8, and cyclo-oxygenase (COX) (69).

**Figure 6:**
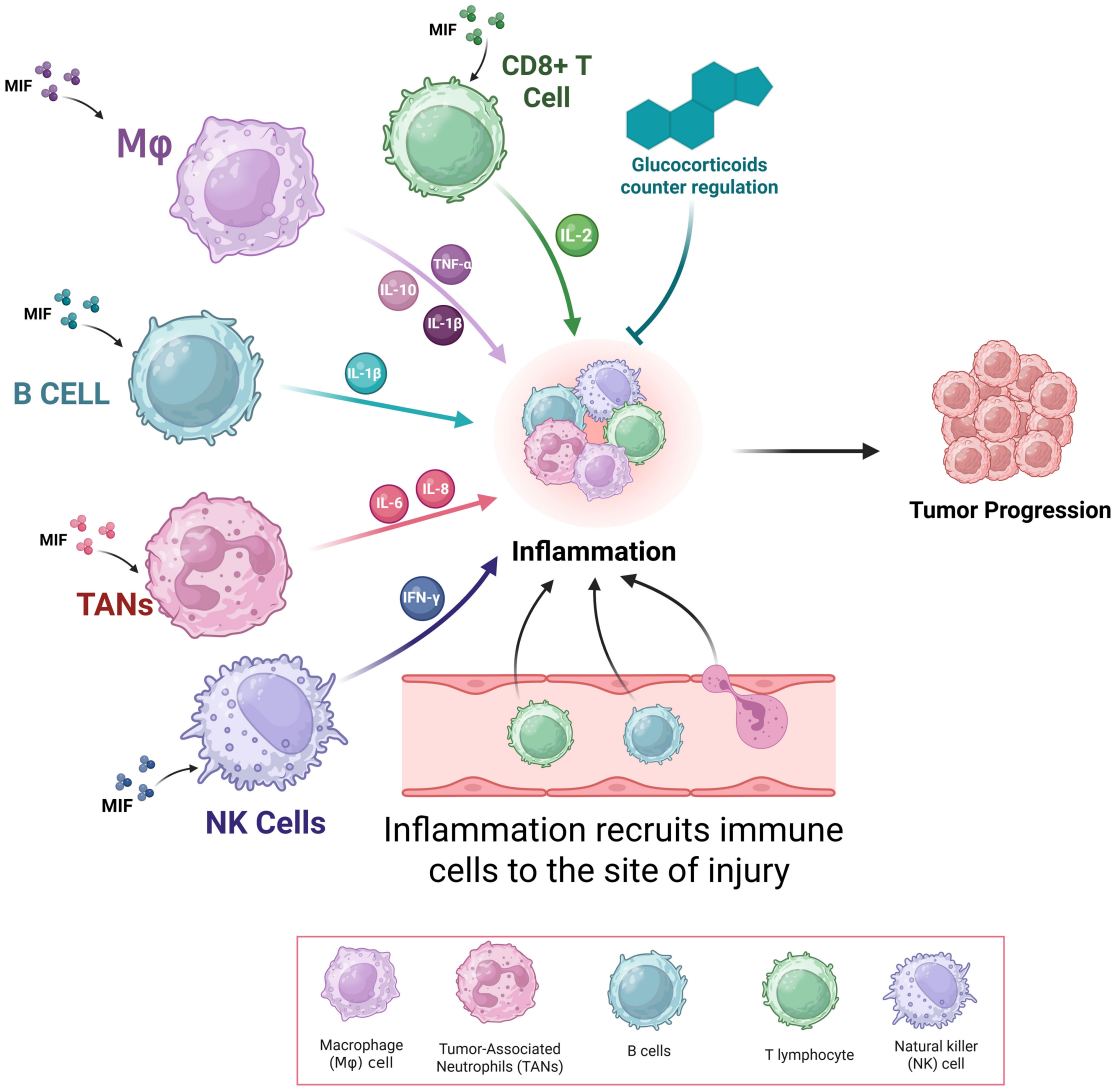
Role of Macrophage Migration Inhibitory Factor (MIF) in Inflammation. *This figure shows the pivotal role played by MIF in the regulation of immune cell function and inflammation. MIF, secreted from immune (T cells, B cells, macrophages, and neutrophils) and non-immune cells, enhances production of pro-inflammatory mediators like TNF-α, IL-1β, IL-6, IL-8, nitric oxide, and COX-2—mainly by macrophage MIF.*.

MIF was initially identified as a product of T lymphocytes; however, studies has shown that endotoxin-induced T cell-deficient mice still exhibit circulating MIF (69). MIF is a pro-inflammatory cytokine and immunomodulator that is rapidly released by both immune cells such as monocytes/macrophages, B cells, and T cells as well as non-immune cells including endocrine, endothelial, and epithelial cells in response to various stimuli as shown in **Figure 6** (3). Growing evidence has revealed that MIF is constitutively expressed in a variety of immune cell types and its over secretion was associated with various pathological conditions. MIF opposes the anti-inflammatory and immunosuppressive effects of glucocorticoids. Normally, glucocorticoids activate MAP kinase phosphatase-1 (MKP-1), which inhibits inflammatory mediators, thereby exerting anti-inflammatory effects (69).

MIF interacts with four membrane receptors: CD74, chemokine receptor with CXC motif 2 (CXCR2), CXCR4, and CXCR7. Depending on the inflammatory context, MIF could bind to individual receptors or receptor complexes, which determine its functional activity (70, 71). This might explain the chemokine-like properties of MIF, thus facilitating the migration and recruitment of leukocytes to sites of infection and inflammation (18, 72).

Once secreted, MIF can act in both autocrine and paracrine manners by binding to these transmembrane receptors, triggering intracellular signaling cascades (73, 74). When MIF binds to CD74, the invariant chain of major histocompatibility complex II (MHC II), it activates signaling through the extracellular signal-regulated kinase (ERK)/MAP kinase pathway and CD44, promoting cellular proliferation and prostaglandin E2 production (16, 75). When MIF binds to CXCR2, it triggers chemotactic responses, driving the recruitment and migration of immune cells, such as monocytes and neutrophils, to sites of inflammation, potentially contributing to the early immune response against infections (18). Similarly, MIF interaction with CXCR4 promotes the directed migration of T cells, playing a role in the adaptive immune response to infections (18). Additionally, the MIF-CXCR7 axis has been implicated in inflammatory processes, particularly in B cell chemotaxis (70).

MIF is primarily secreted by macrophages. A study by Lee et al. demonstrated that autophagy deficient monocytes and macrophages release significantly high levels of MIF when stimulated with LPS (76). Furthermore, MIF plays a crucial role in regulating macrophage responses during infection. It promotes the production of pro-inflammatory cytokines and other inflammatory mediators, including TNF-α, IFN-γ, IL-1β, IL-2, IL-6, IL-8, nitric oxide, and COX2, to facilitate pathogen clearance (3, 77). Macrophage-derived MIF is both sufficient and necessary for driving the angiogenic role of bone marrow-derived macrophages in teratoma formation in mice, highlighting its critical role in promoting M2 macrophage polarization (78). These findings were later validated in mouse models of both primary and metastatic melanoma, where macrophage-derived MIF was essential for the maximal expression of angiogenic growth factors in M2-polarized macrophages and was required for the T-cell immunosuppressive function of melanoma-associated TAMs. Notably, TAMs deficient in MIF or treated with the small molecule MIF inhibitor, 4-IPP, spontaneously shifted to an M1-like polarization profile (79, 80). Collectively, these results demonstrate that loss or inhibition of MIF in solid tumors effectively reprograms intra-tumoral TAMs from an immunosuppressive, pro-angiogenic, tumor-promoting phenotype to an immunostimulatory, non-angiogenic, anti-tumor phenotype (79). Moreover, MIF plays a crucial role in the early innate immune response to parasitic infections, as it facilitates the expression of proinflammatory cytokines and their receptors, promotes parasite recognition, supports macrophage-mediated microbial clearance, and enhances effective antigen presentation (81).

Studies suggest that MIF contributes to immunosuppression by inhibiting cross presentation of tumor antigens by DCs to CD8+ T cells via MHC class I (82). Furthermore, MIF downregulates MHC class II, implying that it also hinders the presentation of tumor antigens to CD4+ T cells (83, 84). Such findings highlight MIF’s role in promoting tumor-associated immune evasion by affecting DC function and consequently CD4+ and CD8+ T cell responses.

Granulocytes such as unstimulated human circulating eosinophils have performed cytosolic MIF. Upon stimulation with phorbol myristate acetate, C5a or IL-5, eosinophils could secrete MIF in a concentration and time-dependent manners (85). Similarly, human neutrophils store preformed MIF, which could be released following secondary necrosis (86). Chen et al. demonstrated that mast cells constitutively express MIF and secrete it during degranulation to modulate innate immune responses, drive fibrogenic activities, and contribute to non-scleroderma-related fibrosis (3, 87). Human eosinophils also possess significant amounts of stored MIF, and elevated MIF levels were detected in the airways of individuals with asthma. In experimental asthma models, mice lacking MIF exhibit reduced pulmonary inflammation and decreased airway hyperresponsiveness compared to their wild-type counterparts (85, 88).

Secreted MIF mediates evasion from NK cell-mediated cytolysis in uveal melanoma, the most common intraocular cancer in adults (89). On the other hand, MIF overexpression was reported to facilitate immune evasion by downregulating NKG2D expression on tumor cells, a key receptor involved in triggering NK-mediated tumor cell cytolysis in ovarian cancer cell lines (90). Other studies reported that MIF may inhibit NK cell function by competing with NK-associated MHC class I molecules (40). Similarly, CTL-derived MIF directly suppressed the anti-tumor activity of primed cytotoxic T lymphocytes (CTLs). Additionally, tumor-derived MIF inhibits T cell activation by inducing T cell death in an IFN-γ-dependent pathway (91).

Another reported mechanism of MIF is the generation and expansion of CD4^+^CD25^+^FOXP3^+^ regulatory T cells by enhancing IL-2 expression in activated splenocytes in mouse models of mammary and colon carcinoma (92). Remarkably, a subset of MIF^−/−^ mice exhibited complete tumor rejection with a significant decrease in Tregs and an increase in CD4+ and CD8+ lymphocytes. Also, tumor-derived MIF enhances the expansion and migration of Th17 cells through CXCR4 signaling. This increase in Th17 cells was associated with improved clinical outcomes (93). Moreover, MIF expression was elevated in advanced stages and more aggressive molecular subtypes of breast cancer, showing a strong correlation with IL-17 levels (94). Beyond cancer, MIF has been linked to IL-17 expression in various autoimmune disorders, such as Hashimoto’s thyroiditis and rheumatoid arthritis, where Th17-mediated responses are known to drive pro-inflammatory processes (95, 96).

The role of MIF in the immune response to microbial pathogens have been extensively studied, revealing that it could either protect the host or exacerbate tissue damage, depending on the specific microorganism involved (29, 97–100). MIF could be protective by activating immune cells, particularly macrophages, and promoting the release of pro-inflammatory cytokines, thus facilitating the efficient clearance of invading pathogens (101–103). Conversely, MIF’s overactivity can exacerbate inflammation, leading to tissue damage. This dual role is particularly evident in chronic inflammatory and autoimmune diseases, where MIF is implicated in sustaining inflammation, resulting in persistent tissue injury and dysfunction (104). In certain viral infections, such as HIV, MIF can create a microenvironment favorable to viral survival and replication, allowing the pathogen to evade immune defenses and establish persistent infections. This detrimental effect may stem from MIF’s ability to dysregulate immune responses, alter cytokine profiles, and increase immune cell susceptibility to viral invasion (105). The impact of MIF is highly context-dependent, influenced by factors such as the specific pathogen, stage of infection, overall immunological environment, and affected organ. Notably, MIF may exhibit organ-specific protective effects in certain diseases, further underscoring the complexity of its role in host-pathogen interactions (106).

Beyond its involvement in infectious diseases, MIF plays a significant role in various inflammatory and pathological conditions, including asthma, atherosclerosis, cancer, autoimmune diseases, burn injuries, and wound healing (107, 108). MIF is implicated in a wide range of autoimmune diseases, reflecting its diverse role in immune regulation (107, 109). High levels of MIF have been observed in patients with SLE, systemic sclerosis, Wegener’s granulomatosis, and relapsing polychondritis (110–112). Elevated levels of MIF have been detected within inflamed synovial tissue of rheumatoid arthritis patients, highlighting its involvement in localized inflammation (113, 114). By counter-regulating the immunosuppressive effects of glucocorticoids, MIF might also contribute to the development of steroid resistance in conditions such as asthma and autoimmune diseases (115).

MIF plays a critical role in amplifying inflammation during carcinogenesis and the development of early-stage hyperplasia or carcinoma as shown in **Figure 6** (116). This is particularly evident in inflammatory colitis, a contributing factor to the progression of colorectal adenomas and adenocarcinomas (117). For instance, in head and neck cancer, elevated MIF levels were associated with increased expression of CD66b, a granulocyte/neutrophil marker, as well as lymph node metastasis, and poorer overall survival (118).

Mechanistically, tumor-derived MIF drives neutrophil chemotaxis via CXCR2 signaling and enhances neutrophil production of CCL4 and MMP9 (118). These MIF-induced factors would promote lymphangiogenesis and tumor-stromal remodeling, thus contributing to tumor progression (119–121). On the other hand, CCL4 can recruit various immune cells, including T lymphocytes, into the TME through CCR5 (121). Thus, the composition and activation state of infiltrating immune cells within the tumor microenvironment would influence whether the response to MIF is pro- or anti-tumorigenic.

## 5 MIF in solid tumors

Over the last two decades, MIF has been linked to carcinogenesis (122), invasion (123), metastasis (124), tumor-induced angiogenesis (125), and disease prognosis and diagnosis (126, 127) of several solid malignancies. The function of MIF in various solid tumors, their diagnostic and prognostic significance, and their potential as a therapeutic target are summarized in **Table 1** and discussed in this section.

**Table 1:** Summarized role of MIF in different solid malignancies.

Cancer	Role of MIFs	Role as biomarkers	Drugs used to target MIFs	Ref.
Head and Neck carcinomas	Play a vital role in cancer development, metastasis, invasion, and deregulation of lipid metabolism	Can be used as diagnostic and prognostic markers (but not in all types)	- Eugenol f - miR-451 - ISO-1	(123, 127–137)
Esophageal cancer	Inducing cancer progression, migration, invasion, proliferation, and angiogenesis	Can be used as prognostic and predictive markers for the effectiveness of treatment	- Anti-MIF Ab	(138–143)
Gastric cancer	Increasing angiogenesis, lymph node metastasis, invasion, growth and survival of cancer cells	Can be used as diagnostic and prognostic markers	miR-1228	(144–150)
Hepatocellular carcinoma	Inducing migration, invasion, immunosuppressive environment, and inhibiting drug-induced apoptosis	Can be used as prognostic and diagnostic markers	- miR-451a - miR-608 - combination between MIF and/or an anti-CD74 antibody and/or the MIF inhibitor ISO-1	(151–158)
Pancreatic cancer	Increasing proliferation, metastasis, immunosuppressive cells, and inhibiting apoptosis	Can be used as diagnostic and prognostic markers	- ISO-1 - 4-IPP - IPG1576	(159–165)
Colorectal cancer	Inducing migration, proliferation, and promoting resistance against MEK inhibitor and oxaliplatin	Can be used as diagnostic and prognostic markers	- 4-IPP -isothiocyanates	(124, 166–171)
Renal cell carcinoma	Promoting tumor development, proliferation and colony formation	Can be used as prognostic markers	- miR-451	(172–175)
Bladder cancer	Increasing proliferation, metastasis, angiogenesis, immunosuppressive environment, and inhibiting apoptosis	Can be used as prognostic and diagnostic markers	- CPSI-1306 - 4-IPP - Anti-MIF Ab	(61, 122, 125, 176–179)
Breast cancer	Inducing proliferation, invasion, migration, promoting M2 macrophages, and increasing ROS production	Can be used as prognostic and diagnostic markers	- CPSI-1306 - siMIF-NP	(180–185)
Lung cancer	Inhibiting apoptosis, increasing angiogenesis, and promoting tumor growth	Can be used as diagnostic markers	- 4-IPP	(79, 186–190)
Melanoma	Playing a vital role in sustaining tumor growth, increasing angiogenesis, promoting metastasis, and inhibiting apoptosis	Can be used as prognostic markers diagnostic markers	- Iso-66 - 4-IPP - Milatuzumab	(191–198)
Glioblastoma	Promoting tumor growth, metastasis, angiogensis and inhibiting the anti-tumor activity of NK cells	Can be used as prognostic markers	- Ibudilast - 4-IPP	(199–203)
Neuroblastoma	Increasing the expression of angiogenic factors, promoting metastasis and migration, and inducing tumor growth	Can be used as prognostic markers	- ISO-1 - 4-IPP - miRNA-451	(204–206)
Ovarian cancer	Increasing tumor progression and inhibiting NK cell activity	Can be used as prognostic and diagnostic markers	- Imalumab	(207–210)
Prostate cancer	Promoting tumor proliferation, metastasis, and invasion	Can be used as prognostic markers	- ISO-1	(211–216)
Cervical cancer	Promoting proliferation, increasing metastasis and migration, inhibiting apoptosis, and disrupting MHC class II antigen presentation, enabling immune evasion			(217–221)
Endometrial cancer	Inducing tumor progression, metastasis, and angiogenesis	Can act as prognostic and diagnostic markers	ISO-1	(222–226)

### 5.1 Breast cancer

Breast cancer (BC) is among the most frequently diagnosed cancers in women (227, 228). MIF plays a crucial role in breast cancer progression and has been the subject of multiple investigations (229, 230). Studies have demonstrated that MIF is significantly overexpressed in non-invasive BC cell lines, such as MDA-MB-468 and ZR75-1, when compared to benign cell lines like MCF-12A. However, MIF expression is notably lower in highly invasive BC cells (MDA-MB-231) even though the MIF receptor CD74 is highly expressed. This suggests that MIF likely targets invasive BC cells through its interaction with CD74. Blocking CD74 has been shown to inhibit MDA-MB-231 cell proliferation, highlighting the role of MIF/CD74 signaling in driving the growth of both non-invasive and invasive cancer cells. Moreover, MDA-MB-468 cells had a 2.1 times higher proliferation rate than MCF-12A cells, yet MDA-MB-231 cells had the highest baseline proliferation rate (approximately 7.8 times higher than that of benign cells). When treated with recombinant MIF (rMIF) at doses in the range of 0–150 ng/ml, MCF-12A and both tumor cell lines demonstrated increased proliferation, with the strongest effect observed in cells with the highest CD74 expression which are MDA-MB-231. MIF not only supports cell growth but also promotes the migration and invasion of cancer cells in a dose-dependent manner. In MDA-MB-231 cells, MIF acts in a chemokine-like manner to facilitate movement through basement membrane-like layers, suggesting that MIF produced in the TME contributes to cancer invasiveness (231). Interestingly, in mouse models, when comparing 67NR non-metastatic cells to both a non-tumorigenic control and the metastatic 4T1 cells, researchers observed higher basal levels of MIF secretion in the 67NR cells. However, this basal MIF secretion was not linked to the invasive phenotype in the studied mouse breast cancer cell lines. Despite the differences in MIF secretion levels, no clear relationship was established between MIF secretion and invasiveness in these models (180). Inhibition of autophagy in 66cl4 triple negative breast cancer cell line led to increased intracellular ROS levels, which further upregulated MIF expression, potentially through ROS-dependent transcription factors. This mechanism underscores MIF’s involvement in macrophage polarization, particularly in promoting the M2 subtype. Research involving MIF-deficient mouse macrophages revealed reduced M1 polarization, suggesting that MIF is essential for macrophage function and amplifies both extracellular and intracellular signaling (181). Additionally, MIF has been identified as a novel 3’ flap nuclease that helps remove unpaired 3’ flaps in DNA during replication. It cooperates with nuclease-deficient polymerases, such as Pol α, to ensure proper DNA elongation and fidelity. In cancer cells, the absence of MIF’s nuclease activity results in increased mutations, slower DNA replication, and cell cycle delays, highlighting its role in helping cancer cells survive DNA replication stress. Moreover, MIF’s recruitment to DNA replication sites by PARP1(Poly(ADP-ribose) polymerase 1) during the S phase further emphasizes its involvement in maintaining cancer cell proliferation (182).

As a biomarker, MIF also has diagnostic and prognostic implications in BC. Immunohistochemical studies have shown that higher MIF levels correlate with larger tumors and more advanced stages of the disease (227). Although MIF expression alone does not significantly affect overall survival, CD163 expression—on tumor-associated macrophages—has been linked to disease-free survival, suggesting that MIF influences the TME in patients with triple-negative BC (183). Furthermore, elevated mRNA levels of CXCR7, an extracellular MIF receptor, have been associated with decreased overall survival, indicating that extracellular MIF signaling plays a critical role in BC progression (181).

Given its wide-ranging physiological and pathological roles, MIF represents a promising therapeutic target for the development of small-molecule inhibitors and antibody-based therapies. In the context of BC treatment, the small molecule CPSI-1306 (isoxazoline drug), which has demonstrated the ability to suppress MIF’s oncogenic activity (184), holds promise as a non-toxic, well-tolerated therapy. CPSI-1306 induces apoptosis in triple-negative BC cells by increasing ROS levels and inhibiting key signaling pathways such as Akt, PDK, and RAF. Its ability to interfere with MIF’s negative regulation of apoptosis further supports its potential use in cancer treatment by promoting cell death while inhibiting proliferation pathways (232). Moreover, a previous study demonstrated the effects of CPSI-1306 treatment and MIF reduction in human TNBC grafts, showing a decrease in the infiltration of MDSCs in the tumor. Additionally, CPSI-1306 treatment appeared to enhance the infiltration of CD8+ T cells, while reducing the levels of granulocyte colony-stimulating factor (GCSF), granulocyte-macrophage colony-stimulating factor (GMCSF), IL-2, and IL-4, when compared to the control group (232). On the other hand, MIF knockdown using siRNA (siMIF-NP) has proven to be an effective method for reducing immunosuppression in tumors. Accordingly, MIF, which is overexpressed in many solid tumors, is a suitable target for siRNA-mediated knockdown. MIF knockdown not only decreases the levels of CD206, a marker for M2 macrophages, but also increases the expression of MHCII, which is crucial for antigen presentation to CD8+ T cells. Accordingly, tumors treated with siMIF-NP exhibit an elevated infiltration of CD8+ T cells, enhancing the immune response and potentially improving therapeutic outcomes (185). Consequently, the therapeutic efficacy of immune checkpoint blockade, a strategy that aids the body’s immune system in attacking tumor cells, was enhanced by targeting MIF (233).

### 5.2 Lung cancer

MIF plays a pivotal role in inflammatory signaling and tumor growth. Its enzymatic function, particularly at the tautomerase active site, is essential for processes like ERK phosphorylation, COX-2 induction, and p53 inhibition, which are crucial for tumor development. Mutation of this active site or inhibition by chemical compounds has been shown to dampen these critical pathways (234). To further understand MIF’s role in lung cancer, researchers explored its interaction with its receptor, CD74, which was found to be widely expressed in lung cancers. In some tumors, CD74 was primarily located in stromal cells, suggesting that MIF might influence the TME. In other cases, CD74 expression was shared between stromal and malignant epithelial cells, hinting at MIF’s involvement in autocrine regulation of angiogenic factors or inhibition of apoptosis in cancer cells. Co-expression of MIF and CD74, detected using factor VII staining, correlated with elevated levels of angiogenic CXC chemokines and greater tumor vascularity, which reinforces MIF’s role in promoting tumor angiogenesis. Inhibition of either MIF or CD74 *in vitro* led to a reduction in the production of these chemokines, further supporting the previous findings (187).

As a biomarker, MIF demonstrates diagnostic potential due to its consistent overexpression innon-small cell lung cancer (NSCLC) tissues compared to normal lung samples. These expression patterns, confirmed by western blotting and immunohistochemistry (IHC), highlight MIF’s overexpression in lung tumors, and its absence in healthy tissues (188). However, its prognostic utility is limited: elevated MIF levels do not correlate with chemotherapy response or overall survival in advanced-stage patients (190). Nonetheless, elevated MIF levels have been consistently associated with increased proliferation and migration of lung cancer cells, reinforcing its potential as part of a diagnostic biomarker panel for lung cancer (189).

On the therapeutic front, MIF inhibition has shown promise. Lungs from MIF-deficient mice showed a noticeable reduction in metastatic tumor burden, both visually and in terms of total lung mass. To assess changes in macrophage polarization, F4/80+ cells were isolated from the lungs of metastatic tumor-bearing MIF-deficient and wild-type mice. Macrophages in MIF-deficient mice exhibited a shift in polarization. The expression of M2 markers, including ARG-1 and IL-10, was significantly elevated, while the expression of M1 markers such as TNF-α was reduced in lung-associated TAMs. Additionally, when 4-IPP was applied *ex vivo* to TAMs from wild-type mice, it caused a similar shift, converting TAMs from an M1-like profile to a more pro-inflammatory M2 profile, further supporting the role of MIF in regulating macrophage polarization (235).

### 5.3 Melanoma

In melanoma cell lines, MIF knockdown led to reduction in the cell number and viability over five days with a notable decline after three days. Western blot analysis confirmed MIF knockdown across six melanoma cell lines, with substantial reductions in Akt phosphorylation in MelCV, Me1007, and MelRMu cells (40–70% decrease), which corresponded with significant cell proliferation inhibition. Notably, melanoma lines resistant to MIF depletion (MelRM and MM200) displayed minimal changes in Akt activity, while the most sensitive lines exhibited the greatest reduction in Akt activity, underscoring a positive correlation between MIF knockdown and Akt signaling (198). These findings demonstrate that MIF plays a significant role in sustaining tumor growth and survival. On the other hand, MIF–CD74 signaling plays a critical role in enhancing melanoma cell survival by promoting a TME favorable to malignancy. Activation of CD74 by MIF contributes to melanoma progression (48), as presented by its influence on immune responses like TNF-α signaling and apoptosis in the TME (197). Additional *in vivo* studies have linked MIF with angiogenesis in melanoma, where anti-MIF antibodies in mice reduced angiogenesis (236). Depletion of MIF also protected mice from lung metastasis by modulating the immune response and reprogramming TAMs, indicating that MIF’s primary role in metastasis is linked to immune system modification (235). Furthermore, high CD74 and MIF ratios correlated with increased pro-inflammatory markers and immune cell infiltration, suggesting that these ratios could serve as important indicators of immune response in melanoma (47).

As biomarkers, MIF and CD74 were identified as key proteins associated with melanoma prognosis. High CD74 and low MIF expression correlated with improved survival outcomes and progression-free survival supporting the idea that the MIF−/CD74+ signature could serve as a prognostic marker in stage III melanoma. This finding is particularly intriguing because these two circulating proteins were able to sort patient outcomes as effectively as tumor-based markers (79). Although results suggest that serum sCD74 may not be a suitable diagnostic marker for early-stage melanoma, its elevated levels in advanced disease suggest a potential role in tumor progression. The increased sCD74 levels could be attributed to secretion by tumor cells themselves or by surrounding cells stimulated by the tumor (196). In addition, poor response to immune checkpoint drugs is substantially correlated with high MIF expression; this is supported by patient data on melanoma that were derived from the TCGA database (198).

Therapeutically, the MIF–CD74 axis presents a promising target in melanoma treatment due to its role in promoting tumor growth and survival. Several compounds have been developed to inhibit MIF signaling (48). Iso-66, a fluorinated oxazoline derivative that has great chemical stability and non-toxic characteristics, was shown to reduce tumor burden in *in vivo* mouse models of melanoma by enhancing antitumor immune responses through the growth of antitumor-specific effector cells. It was also observed in *ex vivo* studies to be associated with the restoration of MIF activity. MIF inhibition suppresses the ability of CTLs and NK cells to target tumors, but reactivating MIF can enhance their antitumor activity (237).

Another inhibitor, 4-iodo-6-phenylpyrimidine (4-IPP), reduced tumor cell proliferation and motility (238), improved survival in melanoma-bearing mice (193) and enhanced the effectiveness of anti-CTLA-4 therapy by increasing CD8+ T-cell infiltration and metabolic reprogramming (194).

MIF-CD74 interaction has been identified as a major factor in preserving a favorable tumor microenvironment for tumor cells and as a regulator of PD-L1 expression. Therefore, a potential target for the efficient treatment of melanoma patients could be the MIF-CD74 interaction (192).

Furthermore, CD74-inhibiting antibodies such as the FDA approved monoclonal antibody milatuzumab can suppress MIF; thus, they can be employed in treatment approaches (191).

### 5.4 Glioblastoma

Glioblastoma (GBM) is an extremely aggressive brain tumor known for its resistance to treatment (239). GBM exhibits marked upregulation of MIF expression compared to lower-grade gliomas (240). This upregulation is accompanied by significantly higher levels of CD74, the MIF receptor, and its co-receptor CD44, as well as non-cognate receptors like CXCR2 and CXCR4. These receptors are notably overexpressed in GBM compared to lower-grade gliomas (241). The pro-tumorigenic role of MIF in GBM is evident through its promotion of proliferation, migration, angiogenesis, and contribution to the immunosuppressive phenotype of glioma cells. High levels of MIF expression are observed in WHO grade IV glioblastomas and high-grade WHO grade III tumors, with the highest levels in the most malignant variants of GBM. MIF is expressed variably in GBM cells, localized primarily on the poles of the nuclei in early-stage cells, and its expression varies with cell passage. Interestingly, while MIF expression ranges between 5.12 and 82.04 ng/ml, it does not correlate with cell proliferation rates, growth behavior, or cell morphology (200). Additionally, MIF signaling through the CD74/CD44 receptor complex activates the ERK MAP kinase pathway, further promoting tumor cell proliferation (203).

MIF negatively regulates wild type p53 signaling in glioblastoma cells, with higher MIF levels associated with functional p53, further implicating MIF in gliomagenesis. Moreover, MIF inhibits the anti-tumor activity of NK cells, reinforcing its role in immune evasion (200).

As a biomarker, MIF shows a complex relationship with glioma prognosis. While treatment groups with elevated MIF expression exhibit poorer prognosis than those with low MIF expression levels (203). However, there is an observed paradox where higher MIF expression does not correlate with reduced overall survival, instead showing a trend toward improved OS. Despite this, neoadjuvant therapy significantly increases MIF expression, further complicating its role in prognosis (241).

Therapeutically, targeting MIF has shown promise in inhibiting pro-tumorigenic traits and restoring immune sensitivity, enhancing the efficacy of radiation and chemotherapy (202). Ibudilast, a drug that penetrates the blood-brain barrier, has been identified as an agent that targets the MIF-CD74 interaction on MDSCs. In preclinical studies, treatment with Ibudilast decreased CD74 expression and increased CD8^+^ T cell infiltration within the tumor, suggesting its potential in treating brain malignancies (242).

### 5.5 Neuroblastoma

By studying AS-MIF-transfected cells, researchers have gained insights into how MIF blockade may affect the expression of molecules related to neuroblastoma. Western blot analysis revealed that AS-MIF transfection significantly reduced N-Myc expression, a gene crucial for neuroblastoma progression. MIF was found in all 21 neuroblastoma samples studied, with both MIF and c-Met (tumor progression related receptors) detected in the cytoplasm of tumor cells. Double immunohistochemistry staining demonstrated that neuroblastoma cells highly expressed MIF and c-Met, with a strong positive correlation between their expressions. Where MIF likely promotes neuroblastoma development by regulating N-Myc and increasing the expression of angiogenic factors. However, transfection of SK-N-DZ neuroblastoma cells with an AS-MIF expression vector reduced MIF expression, leading to a decrease in pro-tumor genes such as N-Myc, TrkB, Ras, and IL-8, while tumor-suppressing genes like EPHB6 and BLU were elevated. In *in vitro* studies and nude mice models, downregulation of MIF significantly reduced neuroblastoma cell proliferation, tumor formation, and metastasis (204).

The role of miR-451 in regulating MIF expression was also studied. Transfection of miR-451 mimics into SK-N-SH and GI-LA-N neuroblastoma cells reduced MIF protein levels, as confirmed by Western blot analysis. A luciferase reporter assay identified a direct interaction between miR-451 and the 3’UTR of MIF transcript, leading to reduced luciferase activity in cells co-transfected with miR-451. This demonstrated that miR-451 negatively regulated MIF expression by directly targeting its transcript. Moreover, the suppression of neuroblastoma cell invasion and migration by miR-451 was reversed when MIF was re-expressed, further supporting MIF’s role as a direct target of miR-451 (243).

As prognostic markers, MIF and its associated receptors play a significant role in neuroblastoma. Analysis of public gene expression datasets revealed that both MIF and CXCR4 were highly expressed in primary neuroblastoma tumors and BM-derived disseminated neuroblastoma tumor cells. An RNA-seq dataset (GSE62564) of 498 primary neuroblastoma samples, was used to evaluate the expression profiles of MIF, CXCL12, and the receptors CXCR4, CXCR2, CXCR7, CD74, and CD44, showed that MIF expression was highest in stage 4 high-risk tumors, while CXCR4 expression was lower in low-risk stage 4S tumor (206).

The biological hallmark of neuroblastomas is the complexity of the genetic abnormalities acquired by the tumor cells. Some of these abnormalities are powerful prognostic markers that are independent of the clinical features (204). Higher MIF or CXCR4 expression was associated with worse overall survival, while elevated levels of CD44, CXCL12, and CD74 were linked to better outcomes. Patients were categorized based on their MIF expression levels, and a survival curve was analyzed for overall survival; patients with lower MIF expression exhibited improved overall survival (206).

Therapeutically, targeting MIF has shown promise in cancer treatment. MIF antagonists, such as ISO-1 and 4-IPP, have been explored as potential therapeutic agents for cancer. They can bind to MIF’s catalytic active site and reduce its activity, leading to decreased tumor growth and extended survival in animal models. However, the cytotoxic effects of these antagonists vary, with ISO-1 showing higher toxicity compared to 4-IPP (206). Moreover a study confirmed that tumor associated MIF suppresses T-cell activation through suppressing three mechanisms of T-cell activation: cytokine, solid-phase anti-CD3,and allogeneic MHC (91). MIF can be targeted by knocking out or inhibitory molecules to restore T cell activation; thus, it can be used as an immunotherapeutic target.

### 5.6 Ovarian cancer

Studies have reported elevated levels of MIF in ovarian cancer (OC) cells, leading to the hypothesis that higher MIF levels may characterize patients with ovarian cancer. Serum analysis from patients with newly diagnosed OC, showed significantly increased MIF levels in the cancer group compared to those in healthy age-matched controls, suggesting a possible link between abnormal MIF expression and ovarian cancer pathogenesis (207). MIF plays a role in immune suppression by inhibiting NK cell function. Specifically, MIF reduces the expression of NKG2D, an activating receptor on NK cells, impairing their ability to lyse ovarian cancer cells. Blocking MIF using anti-MIF antibodies resulted in enhanced lytic activity of NK cells against ovarian cancer cells from ascites. This effect was further confirmed *in vitro*, where NK cells pretreated with supernatant from MIF-depleted ovarian cancer cells showed improved activity against SK-OV-3 cells. In contrast, MIF-rich supernatant reduced NK cell activity.

As a potential biomarker, MIF has emerged as a valuable tool in the prognosis and diagnosis of ovarian cancer. Although MIF levels were not significantly different between patients with early (Stage I/II) and late-stage (Stage III/IV) ovarian cancer (207), higher MIF levels were associated with poor overall survival in recurrent cases, even though MIF had no correlation with progression-free survival (208). These findings suggest that MIF might serve as a valuable biomarker for prognosis in patients with ovarian cancer (209). Furthermore, a proteomic analysis indicated the utility of MIF in diagnostic screening, and a multiplex test combining MIF with other biomarkers, including CA-125 and prolactin, demonstrated high sensitivity (95.3%) and specificity (99.4%) for ovarian cancer detection. In addition, recently a unique multiplex test for six blood biomarkers—prolactin, MIF, insulin-like growth factor II, osteopontin, and CA-125—was developed. This combination of biomarkers appears to exhibit great sensitivity (95.3%) and specificity (99.4%) for the identification of ovarian cancer (209).

Therapeutically, MIF represents a promising target for enhancing anti-tumor immunity. Quantitative real-time PCR and flow cytometry analysis also demonstrated that MIF significantly downregulates NKG2D expression on both NK and CD8^+^ T cells, supporting the hypothesis that MIF contributes to immune evasion by modulating activating and inhibitory signals in these immune cells (52). Thus, MIF could be a potential immunotherapeutic target by reversing its anti-immune effect on NK and T cells. Furthermore, MIF has been targeted therapeutically with monoclonal antibodies like imalumab, which specifically inhibits oxidized MIF. Early clinical trials have shown promising results, with imalumab being well-tolerated and demonstrating stable disease in some patients. Ongoing studies are further exploring its efficacy in patients with ovarian cancer (244).

### 5.7 Prostate cancer

Previous studies highlight the critical role of MIF and its interactions with chemokine receptors CXCR7 and CXCL12 in tumor growth, prognosis, and treatment of prostate cancer. The median MIF serum levels were significantly increased in patients with PCa compared to those in non- PCa individuals (211). This supports previous findings of elevated MIF in patients with CaP (212). *In situ* hybridization of 20 matched benign and malignant prostate tissues showed that MIF mRNA levels were low in benign epithelial cells and confined to the cytoplasm but increased in malignant tissues (211).

Furthermore, the inhibition of the CXCR7 receptor blocked MIF-induced proliferation, highlighting CXCR7’s role in cancer progression. Reducing endogenous MIF levels using siRNA significantly decreased C4-2B cell growth and migration, which could be further suppressed by inhibiting CXCR7 with CCX771. CXCL12 had no effect on cell migration in this PCa model (213).

As a prognostic biomarker, MIF has been implicated in cancer progression and recurrence, particularly in castration-resistant prostate cancer (CRPC). A study of 42 patients with PCa revealed that those with high-expression MIF polymorphisms had a higher recurrence rate (46.2%) compared to those with lower expression (10.3%) within 5 years (214). Additionally, CXCR7 expression was linked to poor prognosis, as patients with high CXCR7 expression had shorter disease-free survival than those with low expression. The related chemokine receptor, CXCR4, did not show a similar correlation with biochemical recurrence, suggesting that CXCR7 plays a more pivotal role in castration-resistant prostate cancer (CRPC) progression (213).

In terms of therapy, MIF raises the concentration of MDSCs in the bloodstream of individuals with diverse malignancies and employs multiple strategies to obstruct NK and T-cell activities, hence reducing anti-tumor immunity (215). Moreover, the malignant cells stimulated by MIF in PCa develop the capacity to eliminate DC by apoptosis and suppress their generation, preventing these cells from acting as antitumor activators. The CXCR7 antagonist, CCX771, and MIF inhibitor, ISO-1, inhibited CaP cell proliferation (213). ISO-1 preferentially targeted DU-145 cells, suggesting that targeting MIF could be a promising therapeutic approach (216).

### 5.8 Cervical cancer

Using immunohistochemistry, MIF expression was examined in surgical biopsy specimens from patients with CC and control subjects. MIF expression was predominantly present in malignant specimens, with most of the expression localized to the cytoplasm of tumor cells (217). similarly, CD74 expression was mainly observed in the cytoplasm (245). Functional assays demonstrate that MIF knockdown (via shRNA) inhibits HeLa cell proliferation and induces apoptosis at early and late stages (218, 219). MIF-CD74 signaling activates oncogenic pathways (e.g., Src, ERK1/2) while dephosphorylating p53 to block apoptosis. CD74 overexpression further disrupts MHC class II antigen presentation, enabling immune evasion and metastasis (220).

As a biomarker, MIF shows limited standalone prognostic utility in CC. Despite its overexpression in malignant tissues, no significant differences in MIF levels or promoter allele frequencies (-794CATT5–7) correlate with tumor differentiation grade (well/moderate/poor) or clinical stage (I/II vs. III/IV) (217, 222). These findings suggest MIF alone is insufficient for risk stratification but may complement multi-marker panels to improve diagnostic sensitivity.

Therapeutically, inhibiting the MIF-CD74 axis holds promise for CC treatment. Preclinical studies show that MIF knockdown reduces tumor cell viability and apoptosis resistance (178, 179). Targeting CD74, overexpressed in CC, could restore MHC class II-mediated antigen presentation to enhance CD4+ T cell activation and block oncogenic signaling (e.g., ERK1/2, p53 suppression) (220). Future strategies should prioritize CD74-directed immunotherapies to counteract immune evasion and tumor survival mechanisms.

### 5.9 Endometrial cancer

MIF plays a significant role in tumor growth and progression by promoting tumor-associated angiogenesis. However, this association has not been clearly observed in endometrial cancer (EC). Research reported high expression of MIF in malignant ascites, and in ovarian cancer cells. MIF enhances the release of cytokines, chemokines, and angiogenesis factors, contributing to tumor vascularization and angiogenesis (246). In EC, MIF mRNA and protein were detectable in all endometrial samples, with upregulation of MIF being associated with early FIGO stages, absence of lymphovascular invasion, and low histological grade. This suggests that MIF overexpression in EC may be linked to the suppression of metastatic spread (247).

In terms of prognosis and diagnosis, research has shown a correlation between MIF concentrations and the prognosis of patients with EC (247). The mean MIF concentrations in the serum of patients with EC (5.871 ± 3.37 ng/mL) were significantly higher than those in healthy individuals (4.825 ± 1.05 ng/mL). ROC curves demonstrated that serum MIF levels could effectively distinguish between EC patients and healthy controls, with an area under the curve of 0.664 (248).

Therapeutically, targeting MIF has shown promising potential in EC. RNAi-mediated knockdown of MIF reduced proliferation and migration in EC cells (224). Additionally, the MIF inhibitor, (S,R)-3-(4-hydroxyphenyl) 4,5-dihydro-5-isoxazole acetic acid methyl ester demonstrated antiangiogenic effects (225). These findings suggest that targeting MIF could be a potential therapeutic strategy in EC (228).

To date, the immunotherapeutic role of MIF in EC is yet to be stated. However, the progression of endometrial tumor cells are thought to be the result of defective NK cells as well as reduced T cell cytotoxicity (249). Previous studies reported the role of MIF on suppressing the anti-tumor effect of NKs (52, 237) and inactivation of T cells (91) in different solid tumors.

### 5.10 Head and neck cancers

Head and neck cancer (HNC) is one of the deadliest cancers worldwide (250, 251). Recent research has illuminated the role of MIF molecules in the progression of this malignancy (126, 252). Increased MIF expression has been observed in head and neck squamous cell carcinomas (HNSCC), specifically in the nasopharygeal carcinoma (NPC) (127), hypopharynx carcinoma (132), laryngopharyngeal carcinoma (253), and oral squamous cell carcinoma (OSCC) (129).

In OSCC, it has been reported that MIF increases the cancer invasion and metastasis via the activation of MMP-2 and MMP-9 (129). Another study found a positive correlation between high MIF expression in OSCC and second primary tumor recurrence (128). Additionally, MIF played role in NPC, the most common malignant tumor of the head and neck carcinomas (130). It was reported that, exosomal MIF derived from NPC promotes metastasis to lung through decreasing and repressing macrophages ferroptosis (130). In another study it was found that, NPC increased in growth and proliferation via MIF/CXCL8 (C-X-C motif chemokine ligand-8)/CXCR2 (C-X-C motif chemokine receptor-2) axis (254).

Moreover, some investigations regarding the role of MIF laryngopharyngeal tumor progression have been conducted (131, 253, 255). A study revealed that signaling axis of MIF-CD44-β1 integrin enhances the spread of laryngeal cancer (255). Another research discovered that hypoxia-induced MIF causes deregulation of lipid metabolism in Hep2 laryngocarcinoma via the IL-6/JAK-STAT (signal transducer and activator of transcription) axis leading to tumor progression (131).

On the other hand, hypopharyngeal squamous cell carcinoma has received very little investigation (132). For instance, it has been reported that increased expression of MIF influences the development and growth of hypopharyngeal squamous cell carcinoma (132).

As a biomarker, MIFs have showed promise as both diagnostic and prognostic biomarkers for head and neck malignancies, with higher levels related with disease presence and severity (126, 134, 256). Its ability to correlate with tumor aggressiveness and patient outcomes demonstrates its value in influencing clinical decision-making and improving patient care (126, 127). For instance, in NPC cells, it was reported that MIF represents a potential non-EBV (Epstein-Barr virus) plasma marker for the diagnosis of NPC (134). In addition, the combination of MIF and the EBV viral capsid antigen antibody (VCA-IgA) has been shown to enhance the specificity and predictive value of detecting NPC and to improve the diagnostic accuracy of NPC in high-risk individuals (134). Furthermore, in a different study, it was found that MIF overexpression in tumor cells is substantially linked to lymph node metastases, advanced clinical stage, and a poor prognosis and so increases its potential to be prognostic biomarker (256). However, a different study found that having a high serum MIF level was associated with a better prognosis and overall survival rate (133). This appears controversial, so further research is necessary.

Additionally, studies showed that MIF could be a potential biomarker in OSCC (135, 257). According to a study, it was revealed that MIF overexpression in OSCC tissues is linked to perineural and deeper tumor invasion as well as lymph node invasion, resulting in a higher pathological stage and low overall survival rate and so it may be a potential marker for poor prognosis (257). Similarly, in another investigation, MIFs high levels have been linked to a worse overall survival rate in OSCC patients, suggesting that they may be a useful prognostic marker for the disease (135).

According to some studies, MIF may also be a useful biomarker for laryngopharyngeal cancers, providing information on the existence and course of the tumor (137). For instance, it was found that patients with laryngeal SCC overexpressing MIF were positively correlated with poor survival and poor prognosis (137).

Current investigations into MIF as a biomarker in hypopharyngeal carcinoma remain limited, highlighting a significant scarce in the existing research and so need more research and investigations.

Therapeutically, there is substantial data regarding MIF as a target in head and neck cancers (131, 136). For instance, Eugenol inhibits the malignant processes of OSCC cells by binding to MIF and decreasing its level (136). In another study it was revealed that ectopic production of miR-451 inhibited NPC proliferation and invasion by targeting MIF mRNA and reducing its expression (123). Moreover, in laryngocarcinomas, it was showed that the MIF inhibitor ISO-1 reduced proliferation and invasion (131). However, there is still a large information gap regarding the use of MIFs as targets in hypopharyngeal cancer, and more investigations are required.

### 5.11 Esophageal cancer

Esophageal cancer is one of most commonly diagnosed cancer and one of the leading causes of cancer death worldwide (258, 259). Similarly, like head and neck cancers, MIFs were found to participate in promoting esophageal squamous cell carcinoma (ESCC) progression (138, 139). According to an investigation, it was found that in ESCC cell lines MIF promotes VEGF and IL-8 production, stimulating the angiogenesis process (143). In another study it was reported that, MIF has been linked to esophageal cancer growth through activating the Akt (Protein kinase B), MEK/ERK (MAPK kinase/extracellular signal–regulated kinase), and NF-κB (Nuclear factor kappa B) pathways, as well as decreasing the expression of the tumor suppressor gene GSK3β (glycogen synthase kinase 3 beta) (139). Moreover, it was found that MIF bind to ACKR3 (atypical chemokine receptor 3) and increases esophageal cancer migration and invasion (138).

As a biomarker, MIF demonstrates clinical relevance for esophageal cancer (140, 142). In a study it was reported that the expression of MIF and its receptor CXCR4 (C-X-C motif chemokine receptor-4) in ESCC was positively correlated with low overall survival rate as so can be used as a prognostic biomarker (140). Another study revealed that MIF expression was negatively correlated with the efficiency of anti-PD-l combined with chemotherapy as a neoadjuvant therapy, suggesting that it could not only be a biomarker for a bad prognosis in ESCC but also a predictive biomarker for the effectiveness of the treatment for ESCC (142).

Therapeutically, targeting MIF remains understudied but shows early promise for esophageal cancer (141). For example, anti-macrophage inhibitory factor 1 antibody has been shown to suppress the growth, proliferation and establishment of the neovascularization lumen of esophageal squamous cell carcinoma in nude mice (141). There is still a great lack of information concerning the use of MIF as a target in esophageal cancer, which calls for more studies and research.

### 5.12 Gastric cancer

Many investigations highlighted the role of MIF in gastric cancer, which considered as one of the most leading causes of cancer-related fatalities (147, 260, 261). For instance, it was found that elevated MIF levels in gastric cancer boosted and improved the angiogenesis process (147). In another study it was reported that, overexpressed MIF in gastric adenocarcinoma increased lymph node metastasis (261). Similarly, it was shown that MIF in gastric cancer increase invasion and lymph node metastasis (148). Another investigation found that the overexpression of ZFPM2-AS1 (ZFPM2 Antisense RNA 1) resulted in upregulation of MIF which led to suppressing the activation and nuclear translocation of the p53 protein which in turn enhanced growth and survival of the tumor cells (146).

As a biomarker, MIF demonstrates diagnostic and prognostic utility for gastric cancer (144, 150). In a study, it was shown that the transcriptome data from CagA+ (cytotoxin-associated gene A protein) gastric carcinomas showed that MIF released in the TME (tumor microenvironment) causes TAM (tumor associated macrophages) polarization, epithelial-mesenchymal transition, and inhibition of MAPK4 pathways—all of which are associated with poor prognosis (144). Another study reported that, elevated levels of MIF were significantly associated with advanced tumor stage, increased lymph node invasion, and poor survival and prognosis, making it a possible prognostic biomarker (150). According to another investigation, it was discovered that high serum MIFs are more sensitive and selective than CEA and hence a better diagnostic marker for gastric cancer (149).

Therapeutically, targeting MIF remains underexplored but promising. In an investigation it was found that, gastric cancer cells’ pro-angiogenic activity is inhibited by microRNA-1228 via binding to MIF and decreasing its expression (145). Further research and investigations are needed to fill up the large knowledge scarce regarding MIF as a therapeutic target in gastric cancer.

### 5.13 Hepatocellular carcinoma

MIFs were also found to have a role in hepatocellular carcinoma (HCC) development, the predominant form of liver cancer (151, 152, 262). According to a study, MIF was found to regulate secreted phosphoprotein 1 (SPP1) and increase TAM migration to HCC cells, and resulted in increased cancer metastasis and invasion (151). In another study, it was shown that MIF suppressed therapy-induced HCC apoptosis (diethylnitrosamine/carbon tetrachloride) and carried out CD74-mediated carcinogenic effects during hepatocarcinogenesis (152). In a single-cell transcriptome study, it was found that CD36+ HCC-associated fibroblasts released MIF through enhanced intratumor lipid oxidation, which improved the immunosuppressive environment by increasing the number of monocytic MDSCs (Myeloid-derived suppressor cells) within tumors (154).

As a biomarker, MIF demonstrates clinical relevance in HCC (153, 157). According to a study, it was found that MIF 755622–2 polymorphism is linked to HCC metastasis and that the GC and CC genotypes were positively correlated with decreased overall survival rate and so can be a predictive marker for poor prognosis (153). In another investigation, it was shown that patients with MIF-794CATT high repetitive-sequence genotypes showed increased metastasis, lower differentiation, and lower survival rate and so it could be a biomarker for poor prognosis (157). Another study reported that plasma MIF was inversely correlated with overall survival rate and had a better value for distinguishing HCC patients from controls (liver cirrhosis, benign lesions, and healthy people). As a result, it may be used as a diagnostic and prognostic marker (155).

Therapeutically, a number of studies suggested that MIFs could be targets in HCC (152, 158). For instance, it was reported that miR-608 decreased HCC progression through directly binding and decreasing MIF expression (158). In another study, it was shown that miR-451a bound to MIF and decreased its expression resulted in inhibiting HCC progression (156). According to another investigation it was found that combination between MIF and/or an anti-CD74 antibody and/or the MIF inhibitor ISO-1 resulted in decreased HCC development and growth (152).

### 5.14 Pancreatic cancer

Investigations have found that MIF also played role in pancreatic cancer development (159, 165). According to a study, it was reported that increased MIF in pancreatic cancer resulted in increased proliferation and metastasis via decreasing P53 expression and translocation which led to decreased low-densitylipoprotein receptor–related protein 1 (LRP1) (165). It was discovered, in a separate research, that MIF tautomerase activity controlled the expression of genes necessary for the recruitment, differentiation, and activation of MDSCs, which led to the build-up of immunosuppressive cells in TME of the pancreatic cancer (160). Moreover, it was shown that MIF helped pancreatic adenocarcinoma cells to evade apoptosis (163). In another study it was found that MIF increased pancreatic cancer progression and metastasis via AKT/ERK (Extracellular signal-regulated protein kinases)/CCND1 (Cyclin D1)/MMP-2 (Matrix metalloproteinase-9) axis (159).

As a biomarker, it was shown that MIFs may have the potential to be biomarkers in pancreatic cancer (159, 161). In a study, it was found that high expression of MIF with correlated with decreased overall survival rate and so associated with poor prognosis (159). Another investigations reported that, serum MIF showed higher levels in pancreatic cancer cells than normal cells and also correlated with poor survival and poor prognosis and so can be a non-invasive diagnostic and prognostic biomarker for pancreatic cancer (161).

Therapeutically, studies showed that MIFs can be a therapeutic target in pancreatic cancer (160, 162, 164). For instance, a study found that MIF inhibitor, ISO-1, decrease pancreatic ductal adenocarcinoma cells proliferation, migration and invasion *in vitro* and *in vivo* (162). Furthermore, in another investigation, it was reported that tautomerase inhibitor, IPG1576 (inhibitor for tautomerase activity of MIF) resulted in decreased exosome-induced MDSCs activation and differentiation and pancreatic tumor growth (160). Moreover, it was found that MIF inhibitor (4-iodo-6-phenylpyrimidine; 4IPP) decreased PANC-1 (pancreatic ductal cancer cells) cells growth and colony formation (164).

### 5.15 Colorectal cancer

MIFs have been frequently described in colorectal carcinoma (CRC), the third most prevalent malignancy and the second-leading cause of cancer death globally (124, 167, 263). In a study it was reported that MIF induces metastasis and growth of CRC cells by targeting SLC3A2 (solute carrier family 3 member 2) and regulating the AKT/GSK- 3β axis (124). Similarly, in another research, it was found that MIF increases proliferation and inhibits apoptosis of colorectal cancer cells (168). Furthermore, it was discovered that KRAS mutant CRC cells treated with refametinib, a MEK inhibitor, stimulated MIF production and resulted in the activation of STAT3 (signal transducer and activator of transcription 3) and MAPK, which promotes resistance to this medication (167). Another study showed that MIF increased oxaliplatin resistance in colorectal cancer by upregulating CXCR7 chemokine (169).

As a biomarker, MIF demonstrates significant clinical utility for CRC (166, 171). MIF (-173 GC/CC) polymorphism, for example, has been demonstrated to be associated with tumor dedifferentiation, advanced illness and poor survival, suggesting that it may be a useful prognostic biomarker for colorectal cancer (171). Another study found that elevated MIF expression in colorectal cancer was associated with an excellent AUC value of 0.933, suggesting that it may be a useful diagnostic marker (166). Lee et al. (170), reported that MIF is a more sensitive but less specific diagnostic biomarker than CEA in early colorectal cancer detection so combination of these two biomarker could be a very useful early diagnostic biomarker.

Therapeutically, targeting MIF shows promise in overcoming resistance and suppressing tumor growth in CRC (167, 264). The combination of refametinib with 4-IPP, significantly lowered STAT3 and MAPK activity compared to single-agent therapy. As a consequence, combination treatment was revealed to have a synergistic growth inhibitory impact against refametinib-resistant colorectal cancer cells via inhibiting MIF activation (167). In a different research, it was demonstrated that isothiocyanates decreased the proliferation of colorectal cell lines via inhibiting MIF tautomerase activity via covalent bonding to the N-terminal proline (264).

### 5.16 Renal cell carcinoma

Renal cell carcinoma (RCC) is the most common type of kidney cancer in adults, representing about 90% of all cases. Its incidence has steadily increased over recent decades, making it a major global health concern (265). Recent studies demonstrate that MIFs were also found to contribute to renal cell carcinoma (RCC) progression (172, 174). According to a study, it was found that MIF knockdown led to decreased proliferation and colon forming ability of renal cell carcinoma (172). Another study found that high MIF expression in the kidney increases RCC development via interacting with HIF1α (hypoxia-inducible factor-1 alpha) and HIF2α (hypoxia-inducible factor-2 alpha) (174).

In terms of diagnostic and prognostic potential, MIF shows promise as a biomarker RCC (173). For instance, high expression in clear cell renal cell carcinoma (one of the most common renal cell carcinomas) was positively correlated with low survival rate and poor prognosis (173). MIF’s potential as a biomarker in renal cell cancer remains largely unexplored and so needs more investigation.

Therapeutically, targeting MIF in RCC has shown early experimental promise (175). According to an investigation, it was found that miRNA-451 decreased and suppressed renal cell carcinoma proliferation, migration and invasion by inhibiting MIF expression via direct binding, highlighting MIF’s potential as a therapeutic vulnerability (175). Despite these findings, the development of MIF-targeted therapies for RCC is still in its infancy, needs more mechanistic studies.

### 5.17 Bladder cancer

In many studies, it was found that MIFs could contribute to bladder cancer progression (122, 179). According to a study, it was reported that MIF can increase proliferation and growth of bladder cancer cells via ERK pathway and also enhances angiogenesis via increasing the expression of VEGF (Vascular endothelial growth factor) (125). In another study it was shown that chimeric transcript SLC2A11 (solute carrier family 2 member 11)–MIF promoted metastasis, proliferation and inhibited apoptosis in bladder cancer cells via PTBP1 (Polypyrimidine tract binding protein)-dependent mechanism (122). Furthermore, it was reported that MIF was increased in muscle invasive bladder cancer and this indicates that MIF could be involved in bladder cancer invasion and migration (179). Another investigation found that, CXCL2/MIF-CXCR2 pathway increased and enhanced recruitment of MDSCs in bladder cancer which produces high immunosuppressive molecules including Arg1 (arginase 1), iNOS (inducible nitric oxide synthase), PD-L1 (Programmed death-ligand 1) and P-STAT3 (266).

In terms of diagnostic and prognostic utility, MIF demonstrates potential as a biomarker for bladder cancer (177, 266). For instance, it was found that Urinary bladder cancer (UBC) was shown to have considerably higher serum MIF levels than urinary bladder disease (UBD) and higher than normal patients indicating that MIF could be a potential diagnostic marker for bladder cancer (177). Another study reported that MIF was negatively correlated with overall survival rate in bladder cancer, highlighting its prognostic relevance (266).

Additionally, MIFs could be potential targets in bladder cancer (176, 178). Anti-MIF antibody reduced the proliferation in HT-1376 cell (human bladder cancer cell) (176). In another study it was reported that CPSI-1306 decreased both tumor development and neovascularization via blocking the enzymatic portion of MIF (125). Furthermore, 4-IPP inhibits and blocks MIF-2 leading to decreased tumor stage and tumor growth (178).

Collectively, these findings establish MIF not only as a driver of tumor progression but also as a promising, though still underexploited, target for therapeutic intervention. Having established the widespread involvement of MIF in the biology of solid tumors, it is essential to consider its potential as a clinical target. The following sections will explore how MIF can be leveraged as a biomarker and a therapeutic target, highlighting both current progress and future opportunities in translational oncology.

## 6 Clinical implications of targeting MIF

MIF has emerged as a promising biomarker across multiple solid tumor types as extensively discussed in the current review. Elevated MIF expression in tumor tissue, serum, or plasma often correlates with advanced disease stage, poor prognosis, and resistance to therapy (155, 267–269). In BC, high MIF levels are associated with more aggressive subtypes such as TNBC (183). Similarly, MIF expression predicts poor survival and increased metastatic potential in colorectal cancer as discussed earlier (270). As a dynamic marker of tumor-immune interactions, MIF levels may also guide patient selection for immunotherapy or combination regimens. Efforts to therapeutically target MIF are ongoing, with several classes of inhibitors under preclinical and early clinical evaluation (271, 272). Strategies include direct enzymatic inhibitors, neutralizing antibodies, and agents that interfere with MIF-receptor interactions as summarized in **Table 2**. Despite promising preclinical results, translation into clinical efficacy remains a challenge, highlighting the need for refined targeting strategies and combination approaches.

**Table 2:** Clinical Targeting Approaches of MIF.

Drug used	Type of the drug	Cancer/disease	Preclinical or clinical phase	Ref.
ISO-1	Small-molecule antagonists targeting tautomerase active site	-Pancreatic cancer -Systemic lupus erythematosus	-Pre-clinical phase -Pre-clinical phase	(162, 273)
4-IPP	Small-molecule antagonists targeting tautomerase active site	Tumor	Pre-clinical phase	(226)
Ibudilast	Small-molecule antagonists acting as allostric inhibitor	Multiple sclerosis	Phase II clinical trial	(271)
Imalumab	Monoclonal antibody neutralizing MIF	Colorectal carcinoma, non-small cell lung cancer, and ovarian cancer	Phase I clinical trial	(191)
BaxG03, BaxB01, and BaxM159	Antibodies that neutralizing MIF and inhibiting MIF-induced phosphorylation	Prostate cancer	Pre-clinical phase	(274)

Small-molecule antagonists targeting MIF have been developed by exploiting its tautomerase active site, which overlaps with the CD74 binding domain (275, 276). Small molecule inhibitors such as ISO-1 and 4-IPP target the tautomerase active site of MIF, disrupting its interaction with receptors and downstream signaling (**Table 2**). 4-IPP forms a covalent bond with MIF, leading to irreversible inactivation (276). While these agents show efficacy in reducing tumor growth in experimental models, issues with specificity and off-target effects have limited their clinical progression. Other small molecule inhibitors block MIF–CD74 binding and show therapeutic activity in mouse models of systemic lupus erythematosus (273). The most advanced MIF antagonist in clinical trials is ibudilast, originally a phosphodiesterase inhibitor, which acts as an allosteric inhibitor of MIF. Ibudilast binds to a dynamic site on MIF that is revealed only upon binding, inducing conformational changes that inactivate MIF (277). It has shown efficacy in a Phase II trial for multiple sclerosis, where high-expression MIF genotypes increase disease risk (271). MIF’s dynamic properties also enable the discovery of agonists, which could be useful in conditions where low-expression MIF alleles are unfavorable.

Developing antibody-based therapies such as monoclonal antibodies that neutralize extracellular MIF or block CD74–MIF interaction is still under investigation (278). A number of monoclonal antibodies targeting MIF have been developed; BaxB01, BaxG03, and BaxM159 induced apoptosis in prostate cancer cells *in-vitro* by inhibiting ERK1/2 signaling, leading to reduced tumor growth in a xenograft mouse model (274, 279). Additionally, Imalumab (Bax69) was evaluated in a Phase 1 clinical trial, which demonstrated promising efficacy against solid tumors such as colorectal carcinoma, non-small cell lung cancer, and ovarian cancer as shown in **Table 2**. Approximately one-third of the patients treated with Imalumab showed stable disease (191). Since anti-MIF antibodies have demonstrated the ability to reduce tumor burden and enhance immune cell infiltration in preclinical studies, such approaches offer improved specificity compared to small molecule inhibitors. However, anti-MIF antibodies still face challenges related to tumor penetration and immunogenicity. Given MIF’s role in immune suppression, combining MIF inhibitors with immune checkpoint blockades such as anti-PD-1/PD-L1 is a compelling strategy. Preclinical studies suggest that MIF inhibition can sensitize tumors to immunotherapy by altering the tumor microenvironment (280, 281). Future clinical trials will be critical to validate these synergistic effects. Despite encouraging advances in targeting MIF therapeutically, significant challenges remain. MIF’s complex and sometimes paradoxical roles in tumor biology, as well as the limitations of current therapeutic approaches, present important hurdles that must be addressed to fully realize its clinical potential.

## 7 Challenges and controversies

Although predominantly characterized as a tumor promoter, MIF may exert context-dependent tumor-suppressive effects in certain context (282). These dual roles complicate therapeutic targeting and necessitate a nuanced understanding of tumor-specific biology (283). Furthermore, MIF’s role varies significantly across tumor types, stages, and even subclones within the same tumor. Factors such as the local immune contexture, presence of specific co-receptors, and tumor mutational burden influence whether MIF acts primarily as a promoter or modulator of cancer progression (284). Thus, personalized approaches to MIF targeting will be necessary to overcome this heterogeneity. In addition, most MIF inhibitors lack tumor specificity and can impact normal physiological processes, leading to potential toxicity. Additionally, compensatory mechanisms, such as upregulation of MIF homologs such as D-DT/MIF-2, may reduce the effectiveness of MIF-targeted therapies (285). Accordingly, there is a pressing need for next-generation inhibitors with improved specificity and for strategies to block redundancy in MIF family signaling. Addressing these challenges requires innovative strategies that account for tumor heterogeneity and the intricate functions of MIF. Emerging technologies and combination therapies offer promising avenues to overcome current barriers, paving the way for more effective and personalized interventions.

## 8 Future perspectives and conclusion

Integrating MIF expressions and signaling profiles into molecular diagnostic panels could help identify patients who are most likely to benefit from MIF-targeted therapies. Genomic, transcriptomic, and proteomic approaches may reveal actionable MIF-driven tumor subtypes, enabling more precision medicine tailored interventions. In addition, combining MIF inhibition with immune checkpoint inhibitors, cancer vaccines, or T-cell therapies holds great promise. MIF blockade may overcome resistance mechanisms that currently limit the success of immunotherapies in “cold” tumors, converting them into “hot” immunologically active tumors. Finally, advanced delivery systems, such as nanoparticle-based carriers, could enhance the precision and efficacy of MIF-targeted therapies. Encapsulation of MIF inhibitors within tumor-targeting nanoparticles may improve biodistribution, minimize off-target effects, and allow combination payloads, such as immunomodulators or chemotherapeutics. As the field continues to evolve, the integration of MIF-targeted therapies into broader cancer treatment paradigms appears increasingly feasible. A deeper understanding of MIF’s biology, coupled with technological innovation, will be critical to translating research advances into tangible clinical benefits for patients. In conclusion, MIF plays a multifaceted role in the progression of solid tumors by promoting inflammation, immune evasion, proliferation, angiogenesis, and metastasis. Its expression correlates with poor clinical outcomes across several major cancers, making it a critical focus for diagnostic, prognostic, and therapeutic research. While challenges remain, including tumor heterogeneity and therapeutic specificity, the future for MIF-targeted interventions is promising. Advances in precision medicine, immunotherapy, and drug delivery technologies may unlock the potential of MIF inhibition as a cornerstone of solid tumor therapy. Continued preclinical exploration and carefully designed clinical trials will be key to translating these insights into meaningful patient benefit.
